# Omics profiling identifies the regulatory functions of the MAPK/ERK pathway in nephron progenitor metabolism

**DOI:** 10.1242/dev.200986

**Published:** 2022-10-03

**Authors:** Hyuk Nam Kwon, Kristen Kurtzeborn, Vladislav Iaroshenko, Xing Jin, Abigail Loh, Nathalie Escande-Beillard, Bruno Reversade, Sunghyouk Park, Satu Kuure

**Affiliations:** ^1^ Helsinki Institute of Life Science, University of Helsinki, Helsinki, FIN-00014, Finland; ^2^ Stem Cells and Metabolism Research Program, Faculty of Medicine, University of Helsinki, Helsinki, FIN-00014, Finland; ^3^ College of Pharmacy, Natural Product Research Institute, Seoul National University, Seoul 08826, Korea; ^4^ Institute of Molecular and Cellular Biology (IMCB), A*STAR, Singapore 138648, Singapore; ^5^ Stem Cells and Metabolism Research Program, Faculty of Medicine, University of Helsinki, Helsinki, FIN-00014, Finland; ^6^ Medical Genetics Department, School of Medicine, Koç University, Istanbul 34010, Turkey; ^7^ GM-unit, Laboratory Animal Center, Helsinki Institute of Life Science, University of Helsinki, Helsinki, FIN-00014, Finland

**Keywords:** Organogenesis, Tissue-specific progenitors, *Pycr1/Pycr2*, Self-renewal, Differentiation, Development, Metabolism, Receptor tyrosine kinase signaling, Intracellular signaling cascades

## Abstract

Nephron endowment is defined by fetal kidney growth and crucially dictates renal health in adults. Defects in the molecular regulation of nephron progenitors contribute to only a fraction of reduced nephron mass cases, suggesting alternative causative mechanisms. The importance of MAPK/ERK activation in nephron progenitor maintenance has been previously demonstrated, and here, we characterized the metabolic consequences of MAPK/ERK deficiency. Liquid chromatography/mass spectrometry-based metabolomics profiling identified 42 reduced metabolites, of which 26 were supported by *in vivo* transcriptional changes in MAPK/ERK-deficient nephron progenitors. Among these, mitochondria, ribosome and amino acid metabolism, together with diminished pyruvate and proline metabolism, were the most affected pathways. *In vitro* cultures of mouse kidneys demonstrated a dosage-specific function for pyruvate in controlling the shape of the ureteric bud tip, a regulatory niche for nephron progenitors. *In vivo* disruption of proline metabolism caused premature nephron progenitor exhaustion through their accelerated differentiation in pyrroline-5-carboxylate reductases 1 (*Pycr1*) and 2 (*Pycr2*) double-knockout kidneys. *Pycr1*/*Pycr2*-deficient progenitors showed normal cell survival, indicating no changes in cellular stress. Our results suggest that MAPK/ERK-dependent metabolism functionally participates in nephron progenitor maintenance by monitoring pyruvate and proline biogenesis in developing kidneys.

## Teaser

**Summary:** A systems biology approach was used to identify the participation of the MAPK/ERK pathway in the regulation of cellular metabolism of nephron progenitors during renal development.

## INTRODUCTION

The differentiation capacity of embryonic nephron progenitors (NPs) is a crucial factor for lifelong renal health as the extent of fetal nephrogenesis determines an individual's final nephron number, and low nephron count predicts a higher risk of renal complications (Luyckx and Brenner, 2010; Bertram et al., 2011). Embryonic kidney development is orchestrated by intercellular and intracellular signaling taking place between two mesodermal derivatives, the ureteric bud (UB) and the metanephric mesenchyme (MM) (Davidson et al., 2019; Kurtzeborn et al., 2019). The process of UB branching both patterns and expands kidney size, whereas the MM hosts NPs, which give rise to all segments of the functional nephron (Costantini and Kopan, 2010). Together, the UB, stromal cells in the most cortical part of the developing kidney and the MM form the NP niche, in which balanced maintenance and propagation of NPs occurs (Oxburgh, 2018). The complex interplay of signals originating from all niche compartments maintains their cell identities without much time-dependent change in niche composition, but with clear differences in cell behaviors and molecular players.

The total NP lifespan is limited to the fetal period in humans and to the first postnatal days of life in mice (Hinchliffe et al., 1991; Hartman et al., 2007; Ryan et al., 2018). The molecular regulation of NP maintenance and differentiation has been extensively studied (O'Brien, 2019; Li et al., 2021a), but the factors contributing to the cessation of the nephrogenic program are only about emerging and suggest involvement of changes in signal transduction activities (Brown et al., 2015; Volovelsky et al., 2018; Li et al., 2021b). Moreover, information is only emerging about the metabolic events and especially mitochondrial metabolism underlying the cell-type-specific behaviors of NPs during their propagation and differentiation switches (Cargill and Sims-Lucas, 2018; Tortelote et al., 2021). A recent study demonstrated higher glycolysis dependance in younger versus older NPs and showed that glycolysis inhibition promotes differentiation, in line with the requirement for aerobic glycolysis (also known as the Warburg effect) in the maintenance of pluripotency (Liu et al., 2017; Shyh-Chang and Ng, 2017). Additionally, Von Hippel–Lindau (VHL) tumor suppressor (*Vhl*) appears to be important for mitochondrial respiration in NPs, which show mild differentiation deficiency in the NP-specific absence of *Vhl* (Cargill et al., 2019).

Although one of the major energy precursors for mitochondria, namely pyruvate, is essential for normal early embryonic development and is suggested to contribute to the cell-fate switches in embryonic stem cells (Conaghan et al., 1993; Butcher et al., 1998; Moussaieff et al., 2015), general understanding of its functions in organogenesis is missing. The last step of pyruvate production is regulated by the embryonic kidney-expressed glycolytic enzyme pyruvate kinase isozyme M2 (PKM2, encoded by *Pkm*); its function is controlled by mitogen-activated protein kinase/extracellular signal-regulated kinase (MAPK/ERK) and its reduced activity has been shown to protect against kidney injury (Yang et al., 2012; Zhou et al., 2019). PKM2, similarly to MAPK/ERK activity, regulates the cell cycle in embryonic cardiomyocytes (Purcell et al., 2007; Maejima et al., 2012; Ihermann-Hella et al., 2014; Magadum et al., 2020).

We have recently demonstrated the importance of MAPK/ERK activity in the transmission of extracellular signals for NP regulation, in which it, on the one hand, maintains NP identity and, on the other hand, propels differentiation in nephron precursors (Ihermann-Hella et al., 2018; Kurtzeborn et al., 2019; Li et al., 2021a). Interestingly, the MAPK/ERK target p53 (encoded by *Trp53*) contributes to metabolic fitness in propagating NPs (Li et al., 2015). The p53 target *Rrm2b*, encoding a catalytic subunit of ribonucleotide reductase (p53R2), is required for nephron function and, interestingly, interacts with the mitochondrial proline synthesis enzymes pyrroline-5-carboxylate reductase 1 (PYCR1) and 2 (PYCR2) (Kimura et al., 2003; Powell et al., 2005; Kuo et al., 2016). Proline metabolism is heavily utilized in cancer cells and deficiencies in its metabolism are causative of many congenital disorders, including different types of cutis laxa with rare renal manifestation (Aguilera et al., 2016; de Koning, 2017; Patriarca et al., 2021). Emerging evidence also supports a regulatory role for proline in the maintenance of pluripotency at least in embryonic stem cells (D'Aniello et al., 2017; Cermola et al., 2021). Single- and double-knockout studies of *Pycr1* and *Pycr2* demonstrated the essential involvement of proline metabolism in neuronal differentiation (Escande-Beillard et al., 2020; Stum et al., 2021), but its other organ- and tissue-specific requirements remain elusive.

We used a systems biology approach to study the cellular functions that MAPK/ERK activity effectuates in NPs. Our combined RNA sequencing and metabolomics characterization of control and MAPK/ERK-deficient NPs revealed cellular metabolism as one of the most important contributing factors in NP regulation. These results establish that the MAPK/ERK pathway guides nephrogenesis in the developing kidney by regulating energy metabolism in NPs.

## RESULTS

### Transcriptional profiling reveals mitochondria and ribosomes as the cellular components most affected by loss of MAPK/ERK activation in nephron progenitors

We recently used a genetic approach to reveal the crucial function of NP-specific MAPK/ERK signaling in progenitor maintenance and propagation (Ihermann-Hella et al., 2018). NP-specific loss of MAPK/ERK activation, achieved by conditional inactivation of both Mek genes *Mapk2k1* and *Mapk2k2* (Six2-TGC^tg/+^; *Mek1*^fl/fl^; *Mek2*^−/−^), resulted in rapid NP deprivation due to cell-intrinsic molecular defects and changes in the niche composition. Transcriptional profiling of MAPK/ERK-deficient NPs isolated from embryonic day (E) 13.5 kidneys (before the onset of significant NP loss) identified over 5000 differentially expressed genes (DEGs) (GSE174229; Kurtzeborn et al., 2022).

**Fig. 1.**
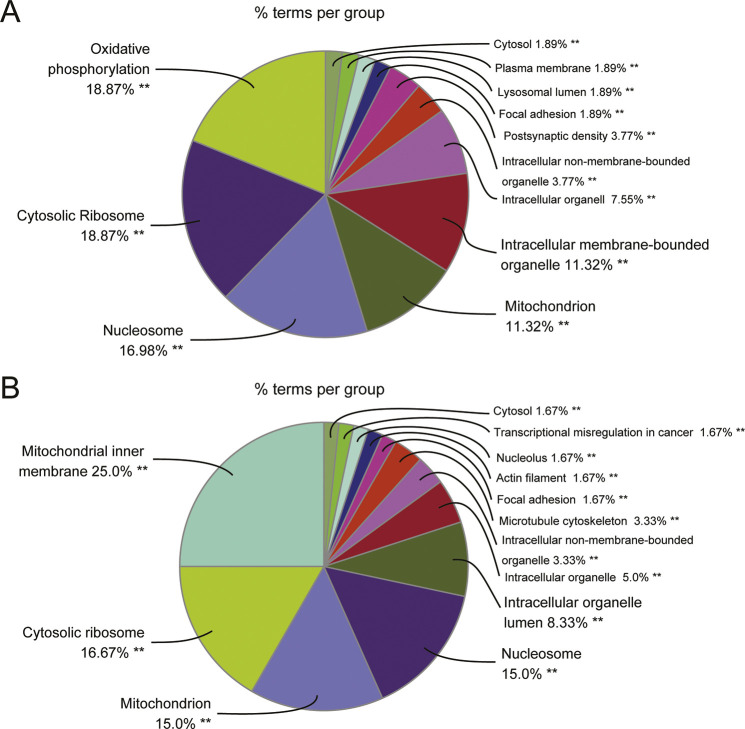
**ClueGO analysis of differentially expressed transcripts in MAPK/ERK-deficient nephron progenitors.** (A,B) Overview pie chart of cellular component and KEGG pathway analyses of the (A) total and (B) downregulated differentially expressed genes (DEGs) between control and MAPK/ERK-deficient NPs. The percentage of genes per term is shown in each group. GO analysis was performed using the Cytoscape plugin ClueGO. The pie chart shows the enriched signaling pathway categories based on the κ coefficient of 0.5. Significantly enriched GO terms are indicate by **P<0.001.

An overview of the gene ontology (GO) analysis of DEGs [*P*_adj_<0.05 and |log_2_(fold change)|≥2] in MAPK/ERK-deficient NPs using the Cytoscape plugin ClueGO (Shannon et al., 2003; Bindea et al., 2009) is listed in Table S1 and shown in Fig. 1A. This illustrates that ‘oxidative phosphorylation’ (18.87%) and ‘ribosome biogenesis’ (18.87%) are the most affected processes. Further characterization of DEGs demonstrated that genes with the GO terms ‘mitochondrial inner membrane’ (25.0%) and ‘mitochondrion’ (15.0%) together account for 40.0% of the downregulated transcripts, which is in line with our previous findings of diminished mitochondrial functions in MAPK/ERK-deficient cells (Kurtzeborn et al., 2022). In addition to energy metabolism, ribosome-encoding transcripts represent the second largest proportion of downregulated genes in MAPK/ERK-deficient NPs (Fig. 1B).

Use of the Kyoto Encyclopedia of Genes and Genomes (KEGG) database (Ogata et al., 1999; Ashburner et al., 2000) revealed fewer pathways than Cytoscape but further strengthened ribosomal contribution, as among the most frequent GO terms were ‘polysomal ribosomes’, ‘ribosomal subunits’, ‘ribosomes’ and ‘NADH dehydrogenase complex’. The upregulated genes were so scattered and few that their pathway analysis failed to provide a meaningful outcome.

### MAPK/ERK-deficiency diminishes the metabolism of amino acids with previous function in stem cell regulation

We next applied a liquid chromatography (LC)/mass spectrometry (MS)-based metabolomics approach to assess MAPK/ERK-affected metabolism in more detail. Reliable metabolomic analysis requires a large number of cells and this presents a challenge due to embryonic NP availability. These cells have been isolated and successfully propagated in nephrosphere cultures, but our pilot experiments revealed that using them for MAPK/ERK-regulated metabolic studies was not feasible owing to the need for extensive growth factors, additional proteins and competitive inhibitors (Brown et al., 2015; Li et al., 2016).

**Fig. 2.**
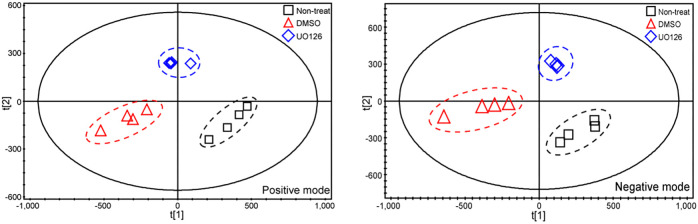
**PLS-DA on LC/MS positive- and negative-mode metabolites identified in mK4 cells upon MEK inhibition.** PLS-DA score plot for mass-to-charge ratios of positive (R²X=0.92; R²Y=0.991; Q²Y=0.968; *n*=1+4 components) (left) and negative (R²X=0.817; R²Y=0.999; Q²Y=0.992; *n*=1+1 components) (right) detection modes for LC/MS. Each symbol represents the metabolic profile of an individual sample in each group (*n*=4). Black squares indicate the non-treated group, red triangles indicate the DMSO-treated group and blue diamonds indicate the MEK inhibitor U0126-treated group. In both detection modes, MEK-inhibited cells, in which MAPK/ERK activation is abolished, share the most similar metabolic profile. R^2^X, total sum of variation in X explained by the model; R^2^Y, total sum of variation in Y explained by the model, representing the overall quality; Q^2^Y, predictive accuracy of the model representing the overall cross-validation co-efficiency.

Guided by the results of RNA-sequencing (RNA-seq) analysis (Fig. 1), we performed targeted metabolomics analysis of the molecules involved in glycolysis and mitochondrial metabolism to quantify principally altered metabolites in an embryonic kidney mesenchyme cell line (mK4). Qualitative measurements of the LC/MS data were analyzed by partial least squares discriminant analysis (PLS-DA), a multivariate statistical technique, which showed clear discrimination for each treatment and control group in both positive and negative detection modes, while clustering well within their treatment group (Fig. 2). This revealed eight statistically different intermediate metabolites upon MAPK/ERK-deficiency induced by treatment with the MEK inhibitor U0126 (Table S2), which include components of glycolysis, the tricarboxylic acid (TCA) cycle and ATP production, supporting our and others’ recent findings that the energy source is an important factor in NP regulation (Li et al., 2015; Kurtzeborn et al., 2022).

Amino acid metabolism is becoming known as an important regulator of different stem cells and their properties (Zhao et al., 2012; Kim et al., 2020; Allmeroth et al., 2021; Someya et al., 2021). To identify the wider spectrum of metabolic changes, an untargeted approach was applied to control and MEK-inhibited mK4 cells. Comparison of the untargeted tandem mass spectrometry (MS2) spectrum patterns with an online MS database revealed a significant decrease of 34 metabolites, including polyamines (N-acetylputrescine), amino acids such as glutamine and its metabolites, L-aspartic acid, leucine, proline, threonine and tryptophan (Table S2) (Melnikov et al., 2016; Krafczyk et al., 2021). Also, methionine, which was recently suggested to contribute to NP proliferation (Makayes et al., 2021), was diminished upon MAPK/ERK deficiency.

**Fig. 3.**
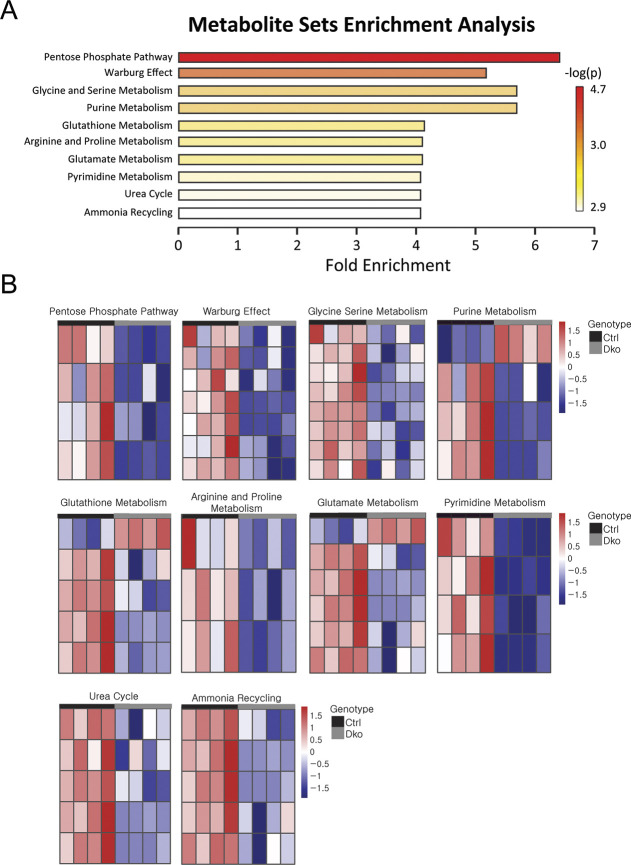
**MSEA and heatmap visualization.** (A) All 46 MAPK/ERK-dependent metabolites identified by LC/MS were applied to MSEA to distinguish the most affected metabolic pathways. The horizontal bar plots are ranked by *P*-values. The color gradient is an indicator of how strongly a metabolic pathway changes, with the strongest changes being red and weakest changes being white. (B) Heatmap analysis of the changes in mRNA expression contributing to the differential pathways identified by MSEA. The individual differential metabolites are listed in Table S3. Each horizontal row, respectively, provides the data for the transcripts as follows: pentose phosphate pathway: D-ribose, R5P, G6P and ADP; Warburg effect: 3-PG, R5P, citrate, ADP, glutamine, G6P and glutamate; glycine serine metabolism: 3-PG, glycine, methionine, ADP, threonine, glutamate, creatine and ornithinine; purine metabolism: xanthine, R5P, ADP and glutamine; glutathionine metabolism: oxidized glutahione, glycine, glutamate, ADP and pyroglutamic acid; argine and proline metabolism: N-acetyl putrescine, creatine and proline; glutamate metabolism: oxidized glutahione, glutamate, ADP, glutamine, glycine and aspartate; pyrimidine metabolism: uracil, ADP, dihydrothymineand glutamine; urea cycle: aspartate, ornithine, glutamate, ADP and glutamine; ammonia recyling: glutamate, ADP, glutamine, glycine and aspartate. Control, Ctrl; double knockout, Dko; G6P, glucose-6-phosphate; 3-PG, 3-phosphoglyceric acid; R5P, ribose-5-phosphate.

Metabolite set enrichment analysis (MSEA) of all identified metabolites by MetaboAnalyst revealed ‘pentose phosphate pathway’, ‘Warburg effect’, ‘glycine/serine metabolism’ and ‘purine metabolism’ as the most altered metabolic pathways in the absence of MAPK/ERK activation (Fig. 3A). Table S3 summarizes the results of the MSEA analysis and lists the contributing metabolites for each category. Heatmap visualization of the metabolite levels accordingly confirmed that metabolic pathways were downregulated in MAPK/ERK-deficient cells (Fig. 3B).

### Integration of metabolomics and RNA-seq data suggests dysregulation in nephron progenitor fate decisions

**Fig. 4.**
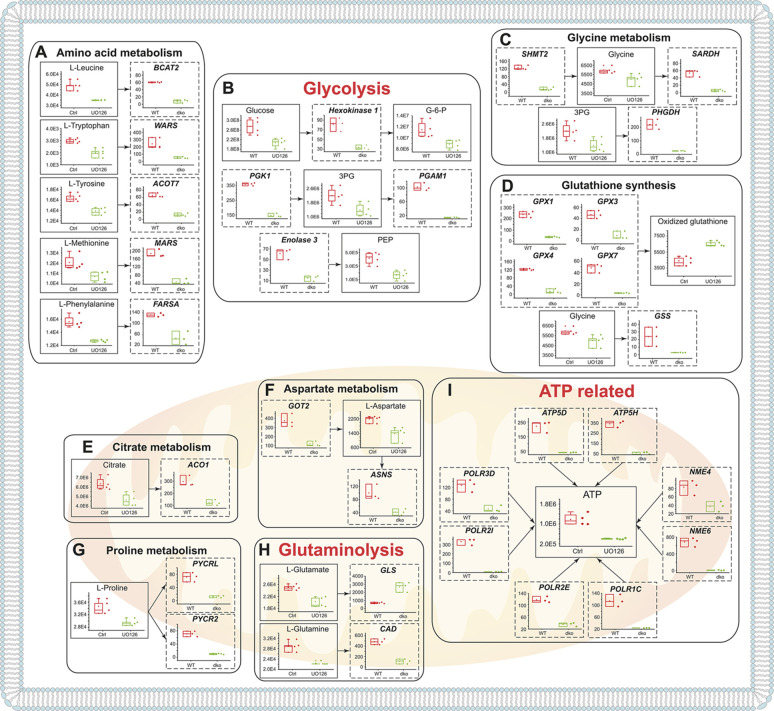
**Integration of identified metabolites and transcriptional level changes of MAPK/ERK deficiency.** (A-I) Quantification of metabolite levels (shown in boxes with solid lines) and gene expression levels (shown in boxes with dashed lines) from metabolomics and RNA-seq analyses, respectively, were used to integrate transcriptional and metabolite level changes. Metabolite levels were measured by LC/MS from four independent control (DMSO-treated) and four MEK-inhibited (U0126-treated) mK4 cell samples. Gene expression level changes were retrieved from RNA-seq performed in isolated populations of three control (WT) and three *Mek1/2* double-knockout (dko) NP populations (GSE174229; Kurtzeborn et al., 2022). For the box plots shown, boxes indicate the 25-75th percentiles, whiskers show the s.d., the central line indicates the average, and each dot represents an individual data point. Dot plots show each datapoint as distinct spots. The *y*-axes indicate detected metabolite levels and relative mRNA expression. (A) These analyses identified diminished levels of the amino acid L-leucine and the related mitochondrially expressed branched-chain aminotransferase 2 (*Bcat2*), which catalyzes the first reaction in the catabolism of essential branched-chain amino acids (including leucine); L-tryptophan and its producer tryptophanyl-tRNA synthetase (*Wars* or *Wars1*); L-tyrosine and acyl-CoA thioesterase 7 (*Acot7*) – acetyl-CoA derived from tyrosine via acetoacetate is catabolized by ACOT7; L-methionine and methionine-tRNA synthetase (*Mars* or *Mars1*); and L-phenylalanine and phenylalanyl-tRNA synthetase subunit α (*Farsa*). (B) Glycolysis-related changes include those in the levels of glucose itself and the levels of the direct downstream modulators hexokinase 1 (*Hk1*) and glucose 6-phosphate (G-6-P); 3-phosphoglyceric acid (3PG) and its upstream regulator phosphoglycerate kinase 1 (*Pgk1*) and downstream metabolic modulators 3-phosphoglycerate dehydrogenase (*Phgdh*) and phosphoglycerate mutase 1 (*Pgam1*); and phosphoenolpyruvate (PEP), the levels of which depends on the activity of enolase 3 (*Eno3*), which also exhibits diminished levels. (C) Reduced glycine levels, together with reduced levels of the enzymes serine hydroxymethyltransferase 2 (*Shmt2*), which metabolizes glycine from serine, and glutathione synthetase (*Gss*) and sarcosine dehydrogenase (*Sardh*), which further metabolize glycine to glutathione and sarcosine, were seen. (D) Glutathione synthesis derives changes not only from glycine shortage (C) but also from reduced levels of glutathione peroxidases 1 (*Gpx1*), 3 (*Gpx3*), 4 (*Gpx4*) and 7 (*Gpx7*). (E) Reduced citrate metabolite levels are associated with reduced levels of aconitate hydratase 1 (*Aco1*), which balances citrate and isocitrate levels. (F) Reduced aspartate levels are associated with lower citrate levels and diminished expression of glutamatic-oxaloacetic transaminase 2 (*Got2*) and asparagine synthetase (*Asns*). (G) Proline metabolism is related to both aspartate metabolism (F) and glutaminolysis (H), and reduced proline levels associate with reduced expression of proline-producing enzymes pyrroline-5-carboxylate reductase family, member 2 (*Pyrc2*) and pyrroline-5-carboxylate reductase-like (*Pyrl*). (H) The levels of glutamate and glutamine metabolites together with glutaminase (*Gls*) and pyrimidine biosynthesis-related carbamoyl-phosphate synthetase 2 (*Cad*), are diminished. (I) Reduced ATP levels associate with several transcriptional changes in ATP synthase subunits [H+ transporting, mitochondrial F1 complex, δ subunit (*Atp5d*) and H+ transporting, mitochondrial F0 complex, subunit D (*Atp5h*)], in RNA polymerase subunits [polymerase I polypeptide C (*Polr1c*), polymerase II polypeptide E (*Polr2e*), polymerase II polypeptide I (*Polr2i*) and polymerase III polypeptide D (*Polr3d*)] and in nucleoside diphosphate kinases (*Nme4* and *Nme6*).

To conduct a more comprehensive analysis at the systems biology level, we next integrated the information from our metabolomics and RNA-seq experiments in different metabolic pathways. The mutually meaningful relationship between the identified LC/MS-based metabolites and RNA-seq gene expression changes was investigated by integrating the results of the two analyses, summarized in Fig. 4.

**Fig. 5.**
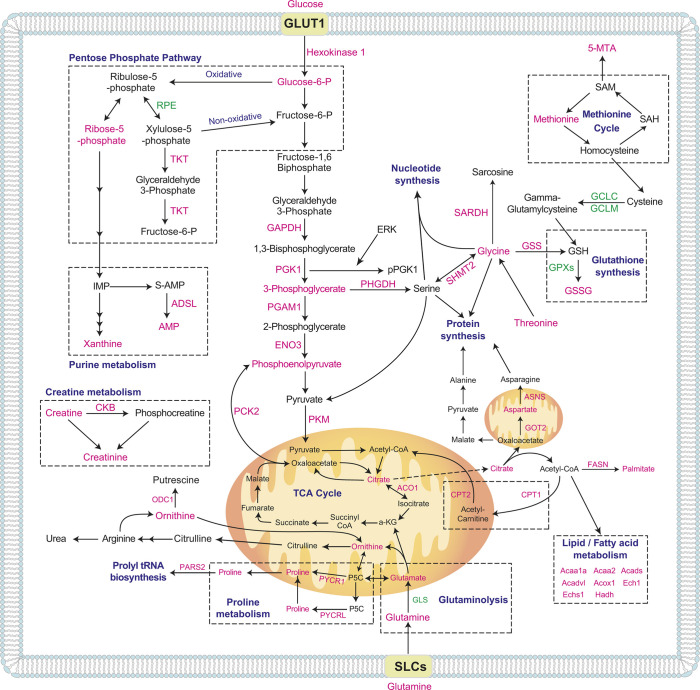
**Presentation of overall pathway map of MAPK/ERK metabolic effects identified by integrating metabolomics and RNA-seq data.** Quantitated metabolites and enzymes are represented in pink (downregulated) and green (upregulated). Dotted boxes represent metabolic pathways including metabolites and genes. 5-methylthioadenosine, 5-MTA; alpha-ketoglutarate, a-KG; acetyl-coenzyme A acyltransferase 1A, ACAA1A; acetyl-CoA acyltransferase 2, ACAA2; acyl-CoA dehydrogenase short-chain, ACADS; acyl-CoA dehydrogenase very long chain, ACADVL; aconitate hydratase 1, ACO1; peroxisomal acyl-coenzyme A oxidase 1, ACOX1; adenylosuccinate lyase, ADSL; adenosine monophosphate, AMP; asparagine synthetase, ASNS; creatine kinase B-type, CKB; citramalyl-CoA lyase, CLYBL; carnitine palmitoyltransferase 1, CPT1; carnitine palmitoyltransferase 2, CPT2; enoyl coenzyme A hydratase 1, ECH1; short-chain enoyl-CoA hydratase, ECHS1;  β-enolase, ENO3; fatty acid synthase, FASN; glyceraldehyde-3-phosphate dehydrogenase, GAPDH; glutamate-cysteine ligase catalytic subunit, GCLC; glutamate-cysteine ligase regulatory subunit, GCLM; glutaminase, GLS; glucose transporter 1, GLUT1; aspartate aminotransferase, GOT2; glutathione peroxidase, GPx; glutathione, GSH; glutathione synthetase, GSS; 3-hydroxyacyl-CoA dehydrogenase, HADH; intermediate of purine metabolism, IMP; ornithine decarboxylase, ODC1; pyrroline 5-carboxylate, P5C; probable proline-tRNA ligase, PARS2; phosphoenolpyruvate carboxykinase 1, PCK2; phosphoglycerate mutase 1, PGAM1; phosphoglycerate kinase 1, PGK1; D-3-phosphoglycerate dehydrogenase, PHGDH; pyruvate kinase, PKM; pyrroline-5-carboxylate reductase 1, PYCR1; pyrroline-5-carboxylate reductase, PYCRL; ribulose-phosphate 3-epimerase, RPE; S-adenosylhomocysteine, S-AMP; S-adenosylhomocystein, SAH; S-adenosyl methionine, SAM; sarcosine dehydrogenase, SARDH; serine hydroxymethyltransferase 2, SHMT2; solute carriers, SLCs; transketolase, TKT.

Among the 46 metabolites we identified, 28 correlated with 113 DEGs (Fig. 4; Table S4) in our RNA-seq data (GSE174229; Kurtzeborn et al., 2022). Although the most significant findings underline mitochondria and energy, diminished amino acid metabolism of tryptophan, glutamine, glycine and proline are supported by *in vivo* transcriptional changes in NP cells (Fig. 4; Table S4). Of these, tryptophan has previously been shown to regulate pluripotent stem cell proliferation and expression of the pluripotency regulator *Oct4* (or *Pou5f1*); glutamine, however, regulates fate reversibility of hair follicle stem cells, and glycine being a component of glutathione functions in the antioxidant response, in one-carbon/folate metabolism with connections to the methionine cycle, and in neuronal specification (Avila et al., 2014; Cheng et al., 2015; Kim et al., 2020; Someya et al., 2021). Interestingly, proline metabolism, which not only regulates stem cell pluripotency but also contributes to the control of neuronal differentiation (Washington et al., 2010; Casalino et al., 2011; Liu et al., 2019; Escande-Beillard et al., 2020; Cermola et al., 2021; Stum et al., 2021), is downregulated in MAPK/ERK-deficient cells, and its direct regulators *Pycr2* (mitochondrial) and *Pycrl* (cytosolic) are also downregulated in MAPK/ERK-deficient NPs. The correlations of metabolite-to-gene expression changes in different pathways are visualized in Fig. 5.

The NP population is composed of cells with heterogenous identities and stem-cell properties based on their differential expression of *Six2*, *Cited1* and *Pax2* (O'Brien, 2019). SIX2^+^ NPs represent a heterogenous population ranging from highly undifferentiated to differentiation-committed cells (Self et al., 2006; Brunskill et al., 2014), whereas SIX2/Cited1 double-positive cells represent the most stem-cell-like and, thus, undifferentiated subpopulation (Brown et al., 2013, 2015).

We next wanted to study whether one of the NP subpopulations would be more dependent on MAPK/ERK activity than the others. To do this, we compared the changes in expression in MAPK/ERK target and metabolism-related genes identified here with previously published single cell RNA-seq (scRNA-seq) results (Brunskill et al., 2014). Analysis of *p53*, a MAPK/ERK target gene present in *Six2*^+^ cells demonstrated 100% expression in scRNA-seq datasets (Table S5). Of the metabolism-related MAPK/ERK target genes, *Pycr1* was present in 44%, *Pycr2* in 77% and *Pkm2* in 100% of the datasets. The expression of differentiation-related genes reflected the known heterogeneity of *Six2*^+^ cells as their expression was sparser among the datasets: *Wnt4* was present in 18.75% and *Lhx1* in 12.5% of the datasets, indicating that some *Six2*^+^ cells are committed to differentiation. This analysis demonstrates that MAPK/ERK targets have higher representation in *Six2*^+^ cells than the differentiation markers.

As expected, *Six2*/*Cited1* double-positive cells showed reduced presence of differentiation-marker-positive datasets for *Wnt4* (16%) and *Lhx1* (8%) than what was identified for *Six2*^+^ cells (Table S5). However, the MAPK/ERK targets *p53* (100%), *Pycr1* (40%), *Pycr2* (80%) and *Pkm2* (100%) either remained highly present (*p53* and *Pkm2*) or demonstrated increased representation in the most stem-cell-like nephron progenitor population. The data together support the conclusions that MAPK/ERK functions in the maintenance of stemness and suggests that control of pyruvate and proline metabolism may participate in progenitor maintenance and propagation.

### Only a few metabolites are increased in MAPK/ERK-deficient cells

Only four metabolites were upregulated in MAPK/ERK-deficient cells (Table S2). These included oxidized glutathione, increased levels of which associate with oxidative stress (Rossi et al., 2006; Tchantchou et al., 2021). Analysis of *in vivo* transcriptional changes in oxidative-stress-related gene signatures revealed downregulation of four major antioxidant enzymes of the glutathione peroxidase family (*Gpx1*, *Gpx3*, *Gpx4* and *Gpx7*), in conjunction with Cu/Zn superoxide dismutase (*Sod1*) and forkhead box 4 (*Foxo4*), encoding a transcription factor regulating reactive oxygen species (Table S6). Additionally, the genes encoding peroxisome biogenesis proteins (*Pex6*, *Pex16* and *Pex10*) and mitochondrial damage responders (*Pink1* and *Atf4*) were downregulated (Table S6) providing a possible link to cellular stress (Schrader and Fahimi, 2006). Interestingly, Parkin (*Prkn*), shown to be transcriptionally activated upon cellular stress (Bouman et al., 2011), was upregulated, along with a modest increase in several other oxidative stress responders including hypoxia-inducible factor 1-alpha (*Hif1a*) (Table S3). These results suggest a perturbed antioxidant defense, which may contribute to the diminished cell cycle progression, which we previously reported in MAPK/ERK-deficient NPs that do not yet show increased apoptosis (Ihermann-Hella et al., 2018).

### Pyruvate affects nephron progenitor niche morphology in a dose-dependent manner

**Fig. 6.**
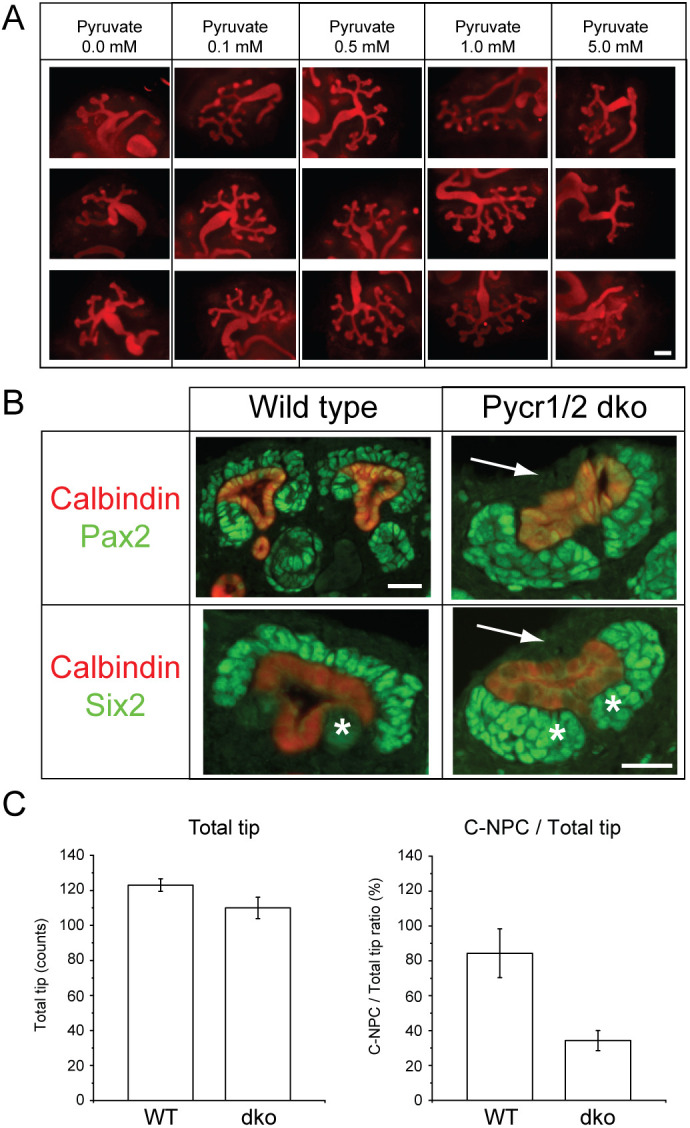
**Functional characterization of identified metabolite effects on nephron progenitor biology.** (A) Embryonic kidneys at E11.5 were cultured for 72 h under different concentrations of pyruvate supplementation and immunostained for calbindin (red) to visualize the entire ureteric bud epithelium. Normal kidney culture medium contained 1 mM pyruvate, which served as a control for other concentrations. Scale bar: 200 µm. (B) P1 control (wild-type) and *Pycr1/2* double-knockout (dko) kidneys were immunostained for PAX2 (green, marker of nephron progenitors and precursors) or SIX2 (green, marker of nephron progenitors) in combination with calbindin (red, marker of ureteric bud epithelium). White arrows indicate the regions where PAX2- and SIX2-positive NP cells should be located but were significantly diminished in the absence of *Pycr1/2*. White asterisks point to the differentiating nephron precursors, which show only very weak SIX2 signal in control kidneys, but to which SIX2 localizes almost exclusively in *Pycr1/2* double-knockout kidneys. Scale bars: 50 µm (calbindin and Pax2 wild type); 200 μm (calbindin and Six2 *Pycr1/2* double-knockout; also applies to calbindin and Pax2 *Pycr1/2* double-knockout and to calbindin and Six2 wild type). (C) Quantification of ureteric tip numbers (total tip) and cortical nephron progenitor cells (C-NPCs) per each tip in the cortex of control and *Pycr1/2* double-knockout kidneys (*n*=2 kidneys/genotype, a total of 15 randomly selected medullar sections were counted). Data are average±s.d.

To investigate the physiological consequences of the identified metabolic changes, we first assayed the effect of pyruvate on early kidney development. E11.5 kidneys were cultured for 72 h *in vitro* as described earlier (Ihermann-Hella and Kuure, 2019) under normal (1 mM) and different pyruvate concentration conditions. Cultured kidneys were immunostained for calbindin to visualize ureteric bud tips, which form an extrinsic niche for NPs (O'Brien, 2019) and branching morphogenesis (Fig. 6A). Without pyruvate, kidney growth through branching morphogenesis was severely compromised (50-80% less tips), the trunks were abnormally long, and the tips failed to enlarge, especially with less than 0.5 mM pyruvate concentrations. We and others have previously published the effect of MEK inhibitor(s) on ureteric bud branching morphogenesis (Fisher et al., 2001; Hida et al., 2002; Ihermann-Hella et al., 2014). Moreover, our ureteric bud-specific genetic experiments demonstrate a very similar phenotype to that observed with chemical MEK inhibition (Ihermann-Hella et al., 2014). In essence, MAPK/ERK-deficient ureteric buds fail to generate complex branched epithelial structures. This is due to failure of ureteric bud tips, which contain the collecting duct progenitors, to expand and form an enlarged ampulla preceding the tip bifurcation required for new branch formation. The MAPK/ERK-deficient morphology resembles the ureteric bud morphology observed here in low pyruvate concentrations (Fig. 6A), thus supporting our findings that MAPK/ERK regulates progenitors by contributing to their metabolic control. Interestingly, pyruvate concentration higher than 1 mM resulted in diminished branching and tip amounts but with a different pattern than in low concentrations (0-0.5 mM) and without a dramatic effect on tip morphology, further confirming the role of metabolism in the regulation of kidney progenitors.

### Proline metabolism is required for nephron progenitor maintenance

Our integrated metabolomics and transcriptomics data revealed downregulation of proline metabolism, which is of particular interest as, in addition to reduction of the metabolite itself, the expression of its regulatory genes *Pycr2* and *Pycrl* was significantly diminished in MAPK/ERK-deficient NPs (Fig. 4).

The publicly available GUDMAP database (https://www.gudmap.org/) shows high *Pycr1* expression in the developing kidney from E11.5 onwards. Based on microarray analysis, *Pycr1* expression is highest in the NP cells and in their early differentiating descendants. The Genepaint database reveals a more restricted expression pattern for *Pycr2*, which, however, seems to overlap with *Pycr1* in differentiating nephron precursors (https://gp3.mpg.de/viewer/setInfo/EH1140/10). To assess the physiological *in vivo* requirement of proline metabolism in kidney development, we utilized the conventional inactivation of *Pycr1* and *Pycr2* genes in mouse. Inactivation of both *Pycr* genes resulted in Mendelian ratios of homozygous single knockouts of *Pycr1^−/−^* and *Pycr2^−/−^* as well as double-knockout *Pycr1^−/−^;Pycr2^−/−^* pups. Characterization of *Pycr1^−/−^;Pycr2^−/−^* mouse kidneys at postnatal day (P) 1 revealed no abnormalities in size and morphology based on histological examination (Fig. S1A-C).

We next analyzed the NP population and nephron differentiation in *Pycr1/Pycr2* (hereafter *Pycr1/*2) double-knockout kidneys. Visualization of PAX2-positive cells, which represent the ureteric bud, NPs and nephron precursors of the developing kidney (Naiman et al., 2017; Grimley and Dressler, 2018), suggested defective NP maintenance in the *Pycr1^−/−^;Pycr2^−/−^* postnatal kidneys (Fig. 6B). SIX2 expression, however, has been shown to be high in the NPs throughout fetal development but, before the final loss of NPs in postnatal wild-type (WT) kidneys at P3, it shifts to localize more abundantly in nephron precursors (Kobayashi et al., 2008; Rumballe et al., 2011). Markedly reduced numbers of SIX2-positive NP cells were detected per ureteric bud in *Pycr1^−/−^;Pycr2^−/−^* postnatal kidneys than in control kidneys (Fig. 6B,C), demonstrating NP cell dependence on proline metabolism.

**Fig. 7.**
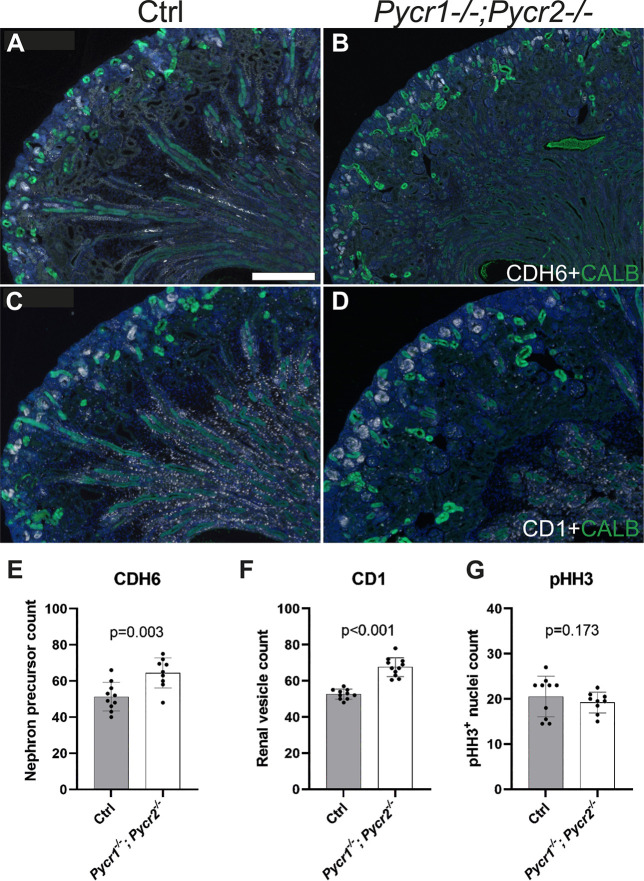
**Nephron progenitors are prematurely lost in the postnatal kidneys of *Pycr1/2* double-knockout mice.** (A,B) Representative images of P1 (A) control and (B) *Pycr1/2* double-knockout kidneys, which were co-immunostained with cadherin 6 (CDH6, white) to visualize epithelializing nephrons from early pretubular aggregates to S-shaped bodies, and calbindin (CALB, green), highlighting the ureteric bud epithelium. (C,D) Representative images of immunostaining in P1 (C) control and (D) *Pycr1/2* double-knockout kidneys in which cyclin D1 (CD1, white) staining indicates differentiation-committed nephron progenitors in the renal vesicles and calbindin (CALB, green) highlights the ureteric bud epithelium. Scale bar: 200 µm. (E-G) Quantification of (E) cadherin 6-positive and (F) cyclin D1-positive differentiating nephrons as well as (G) overall proliferation [phospho-histone H3 (pHH3) was used as a mitotic marker] in control and *Pycr1/2* dko kidneys (*n*=3 kidneys/genotype, 3-5 randomly selected medullar sections were counted). Data are average±s.d. A Mann-Whitney test was used to determine significance.

To gain mechanistic understanding for premature NP decrease in *Pycr1^−/−^;Pycr2^−/−^* kidneys, we hypothesized the involvement of three contributing mechanisms: increased proliferation, decreased apoptosis or accelerated differentiation. The possible involvement of proliferation and/or apoptosis was analyzed by visualizing mitotic cells with phospho-histone H3 and apoptotic cells with cleaved-caspase 3, and these analyses showed no differences between control and *Pycr1^−/−^;Pycr2^−/−^* kidneys (Fig. 7G and Fig. S1D-G). Analysis of mitochondrial morphology revealed no alterations in NPs of *Pycr1^−/−^;Pycr2^−/−^* kidneys (Fig. S2).

We then performed immunofluorescent staining for cadherin 6 (CDH6), a nephron differentiation marker, to visualize the bulk of differentiating nephrons ranging from early pretubular aggregates to late S-shaped bodies, followed by a quantitative analysis, which demonstrated an increase in nephron precursors (Fig. 7A,B,E). We next used another differentiation commitment marker, cyclin D1 (CD1, encoded by *Ccnd1*), which detects renal vesicles that represent a more restricted precursor pool with a more defined and earlier differentiation status (Fig. 7C,D). Indeed, CD1 staining revealed a statistically significant increase in early nephron precursors of kidneys with defective proline metabolism without effects on proliferation (Fig. 7F,G), supporting that the earlier initiation of the final burst of nephrogenesis begins in *Pycr1*^−/−^; *Pycr2*^−/−^ kidneys. Taken together, these results demonstrate that proline metabolism, which, in this study, was identified to depend on MAPK/ERK activation, is essential for preventing premature differentiation of the postnatal NPs, through which it contributes to progenitor maintenance.

## DISCUSSION

Systems biology is emerging as a powerful approach to study functional linkages of metabolic and transcriptional changes (Kitano, 2002; Pang et al., 2019). Metabolomics has the potential to link genotype to phenotype (Fiehn, 2002), and it has been broadly and successfully used in systems biology studies (Weckwerth, 2003) to gain a better understanding of complex biological systems, as exemplified by metabolomics studies of mitochondrial dysfunction in kidney diseases (Nam et al., 2009; McKillop and Flatt, 2011; Atzler et al., 2014; Sharma et al., 2013; Tran et al., 2016). We have previously demonstrated the importance of the MAPK/ERK pathway in NP maintenance and differentiation during embryonic kidney development (Ihermann-Hella et al., 2018). Here, we combine metabolomics and transcriptomics characterization of MAPK/ERK-deficient NP cells to advance the understanding of cellular mechanisms contributing to kidney development.

Congenital anomalies of the kidney and urinary tract (CAKUT) are common birth defects caused by abnormalities in kidney morphogenesis and contribute to the worldwide burden of kidney diseases (Anand et al., 2014; Luyckx et al., 2018; Jain and Chen, 2019). The CAKUT phenotype spectrum varies from mild renal hypoplasia with reduced nephron count to hypodysplasia and a complete lack of kidneys (Murugapoopathy and Gupta, 2020). The milder CAKUT forms, such as reduced nephron mass due to impaired NP maintenance or differentiation, are not life-threatening at early ages but increase the risk of chronic kidney disease with cardiovascular comorbidities later in life (Luyckx and Brenner, 2010; Bertram et al., 2011). As the final nephron count in each individual is determined by renal morphogenesis during fetal development, any factor possibly affecting the rate and extent of nephrogenesis is of high interest.

Our results indicate that MAPK/ERK-deficiency affects both pyruvate and proline metabolism, which are interconnected through glycolysis and glutaminolysis (Fig. 5). Pyruvate, an end product of glycolysis, is converted into acetyl coenzyme A (acetyl-CoA) to feed the mitochondrial TCA cycle. Proline is mainly synthesized from glutamate during glutaminolysis (De Ingeniis et al., 2012), which has crucial compensatory functions under glucose-deprivation conditions owing to its ability to convert glutamine into glutamate (Wu et al., 2014; Yang et al., 2014). As glycolysis and glutaminolysis are the main energy metabolism pathways in mammals, they both likely contribute to energy deprivation in MAPK/ERK-deficient cells.

The production of pyruvate requires the function of the embryonic kidney-expressed and MAPK/ERK-regulated glycolytic enzyme PKM2 (Gupta and Bamezai, 2010; Yang et al., 2012). Our results demonstrated decreased expression of *Pkm2* and reduced levels of the TCA intermediate citrate in MAPK/ERK-deficient cells. Effects of different pyruvate dosages showed dose-dependent changes in the morphology of ureteric bud tips, which serve as an extrinsic niche for NPs (O'Brien, 2019). This suggests that low pyruvate availability, for example, due to diminished conversion of precursor phosphoenolpyruvate to pyruvate, supports ureteric bud growth and elongation, but fails to generate new tip niches, which are essential for NP maintenance (Li et al., 2021b). High pyruvate concentrations reduced the number of tip niches as well but without significant effects on tip morphology. Interestingly, forced redirection of pyruvate to mitochondrial acetyl-CoA production increases pluripotency, whereas glycolytic acetyl-CoA production drives histone acetylation in embryonic stem cells (Moussaieff et al., 2015), supporting a crucial role for pyruvate availability in NP stemness regulation.

Proline metabolism has versatile functions in cellular homeostasis (Patriarca et al., 2021) and its role as a stem-cell regulator is of high relevance to our study. Proline supplementation to embryonic stem cells induces their proliferation and induction towards epiblast fate, whereas maternal proline improves nutrient transport to better support pre-implantation embryo development (Washington et al., 2010; Casalino et al., 2011; Cermola et al., 2021; Someya et al., 2021). Proline deficiency causes several different inherited diseases that typically affect growth, the central nervous system, and renal functions (Patriarca et al., 2021).

Our *in vivo* characterization of *Pycr1/2-*deficient kidneys revealed the importance of proline metabolism for NP maintenance in the postnatal kidney. Our results show that in the developing kidney, *Pycr2* can compensate the loss of *Pycr1*, in accordance with the recent report on their genetic interactions (Stum et al., 2021). Interestingly, although PYCR1-deficiency, which in humans causes cutis laxa, causes cell death in the mouse central nervous system and in *Xenopus* and zebrafish skin (Reversade et al., 2009; Kuo et al., 2016; Escande-Beillard et al., 2020), *Pycr1/2*-deficient kidneys did not show increased apoptosis. Instead, the loss of the enzymes involved in the last steps of proline biosynthesis induced premature differentiation in NPs of the postnatal kidneys. Identification of proline as one of the regulators of NP cells is an important result, not only because the exact functions of proline metabolism at the organ and tissue levels have remained unknown, but also owing to its therapeutic potential as a metabolite that is easy to administer.

It has been shown that under pyruvate deprivation, glutaminolysis increases the concentration of mitochondrial glutamate, which can be converted into pyrroline 5-carboxylate (P5C), an intermediate metabolic product that contributes to both the biosynthesis and catabolism of proline. As proline is mainly synthesized from glutamate or ornithine (Jones, 1985), decreased levels of mitochondrial glutamate due to poorly functioning glutaminolysis may directly affect P5C production and contribute to defective proline synthesis in MAPK/ERK-deficient cells (Fig. 5).

In this study, we focused on understanding MAPK/ERK-dependent transcriptional and metabolic control of NP cell maintenance, propagation and differentiation. For this, NP cells with intact and depleted MAPK/ERK activity were used. This strategy is a powerful way to study NP niche-intrinsic mechanisms of nephrogenesis guidance. However, it bypasses the biological complexity of whole-kidney morphogenesis, which depends on the functions of the ureteric bud epithelium and stroma. We and others have shown that several ureteric bud-derived signals significantly contribute to NP cell biology (Karner et al., 2011; Lindström et al., 2015; Li et al., 2021b) and the regulatory function of stromal signals is emerging (Naiman et al., 2017; Zhang et al., 2019). Thus, further studies are needed to build up a holistic view of how NP metabolism is integrated in the control of an optimally functioning kidney.

## MATERIALS AND METHODS

### RNA isolation for sequencing and data analysis

For sequencing, RNA was isolated from nephron progenitors sorted by fluorescence-activated cell sorting at E13.5 using a standard protocol for chloroform/isopropanol extraction, followed by DNase I treatment according to the manufacturer's instructions (Thermo Fisher Scientific, EN0521). Total RNA quantity and quality were estimated using the Bioanalyzer RNA Total Pico (Agilent Technologies) and NanoDrop spectrophotometer analysis. Library preparation was done using NuGen Ovation Solo (https://lifesciences.tecan.com/ovation-solo-rna-seq-library-preparation-nuquant?p=tab--1) and sequencing was performed with NextSeq 500 (Illumina) at the BIDGEN DNA Sequencing and Genomics Laboratory (University of Helsinki). To convert BCL files to FASTQ files and demultiplex samples, bcl2fastq2 Conversion Software was used (Illumina). Sequenced reads were trimmed for adaptor sequence and masked for low-complexity or low-quality sequences using Trimmomatic (Illumina). Trimmed reads were mapped to the GENCODE *Mus musculus* release M23 reference genome GRCm38 using STAR aligner (2.6.0c) (https://www.gencodegenes.org/mouse/release_M23.html).

Raw gene counts were normalized using the DESeq2 R package (v.3.6.2) and sample clustering was visualized by principal component analysis. Multiple testing adjustment of *P*-values was done using the Benjamini–Hochberg method to compare gene expression levels in three control and three nephron-specific MAPK/ERK-deficient samples (*Six2-TGC*, *Mek1^fl/fl^* and *Mek2^−/−^*). The RNA-seq raw data is available at GEO (GSE174229; Kurtzeborn et al., 2022).

### Gene Ontology and pathway analysis

To analyze the differing metabolic characteristics of MAPK/ERK-deficient NP populations, we used our recent whole-genome RNA-seq data (GSE174229; Kurtzeborn et al., 2022). To interpret DEGs in NPs, we performed pathway analysis using the Cytoscape plugin ClueGO (https://apps.cytoscape.org/apps/cluego) to create a functionally organized gene ontology. The GO Cellular Component and KEGG ontology were used for GO analyses, and we performed the GO analyses using total DEGs and upregulated and downregulated DEGs separately.

### Sample preparation for metabolomics

To investigate metabolic changes in NPs caused by MEK inhibition, we analyzed metabolic changes in the mK4 cell line, derived from embryonic kidney mesenchyme, through a LC/MS-based metabolomics approach. Prior to cell harvesting for metabolite extraction, mK4 cells were incubated with dimethyl sulfoxide (DMSO, 0.1% v/v) or MEK inhibitor U0126 (15 µM, Sigma Aldrich, 9903), or cultured without treatment. After 24 h incubation, cells were harvested using 0.25% trypsin/EDTA (Gibco, 25200056) treatment and washed with ice-cold PBS. The metabolites were extracted using the methods described in Kurtzeborn et al. (2022). In brief, harvested cells were dissolved in 600 µl of methanol/chloroform solvent (2:1 v/v), flash frozen twice with liquid nitrogen and thawed at room temperature. Then, 200 µl chloroform and 200 µl distilled water were sequentially added, and metabolites were separated by centrifugation at 15,000 ***g*** for 20 min at 4°C. Finally, water-soluble metabolites (upper phase after centrifugation) were dried using a centrifugal vacuum evaporator. The protein aggregate (middle phase) was isolated and dissolved in 6 M urea buffer, and total protein concentration was measured for normalization.

### LC/MS analysis

Chromatographic separation was performed on a ZIC-HILIC column (3.5 µm, 200 Å, 100×2.1 mm; Merck Millipore) using an Acquity UPLC system (Waters, MA, USA). The column temperature was 35°C with a flow rate of 0.4 ml/min and the auto-sampler cooler temperature was 4°C with an injection volume of 2 µl. Analytes were eluted with a mobile phase composed of 10 mM ammonium acetate in water with pH 6.8 (buffer A) and 10 mM ammonium acetate in water:acetonitrile solution (1:3) with pH 6.8 (buffer B). The gradient conditions were as follows: 0-1 min, 100% buffer B; 1-7 min, decreasing concentration of buffer B from 100% to 20%; 7-11 min, 20% buffer B, 11-23 min 100% buffer B. MS experiments were performed on a Q Exactive Focus Orbitrap Mass Spectrometer (Thermo Fisher Scientific) equipped with heated electrospray ionization sources. To detect as many ions as possible, both positive and negative detection modes were used in the LC/MS experiment. The inclusion list for parallel reaction methods is shown in Table S7. The MS2 spectrum patterns were compared with the online MS database (Wishart et al., 2018). The METLIN database and high-resolution m/z from the full MS experiment were applied to confirm target metabolites (Guijas et al., 2018). All the measured data were normalized against total protein concentration.

### Metabolomics analysis and systems biology

Multivariate statistical analysis was carried out using SIMCA-P (Umetrics, Umeå, Sweden; v. 11.0). To distinguish metabolic pattern differences from each experimental group, the supervised statistical method PLS-DA was performed using the processed data from LC/MS positive and negative modes, and the metabolites contributing to separation on PLS-DA were identified. To further analyze the identified metabolites, we carried out MSEA using a web-based tool from MetaboAnalyst (Xia and Wishart, 2010), and the levels of individual contributing metabolites from the MSEA results were visualized by heatmap analysis in R.

### Mouse lines

*Six2-TGC*, *Mek1^fl/fl^* and *Mek2^−/−^* mice and their genotyping have been described previously (Scholl et al., 2007; Kobayashi et al., 2008; Belanger et al., 2003). Mice were kept in mixed genetic backgrounds containing C57BL6/Rcc and 129/SvEv strains. The kidneys were dissected at indicated stages according to a published protocol (Ihermann-Hella and Kuure, 2019). Animal husbandry and procedures were approved by EU legislation and the Finnish Animal Care and Use Committee.

### Effect of pyruvate on embryonic kidney culture

E11.5 wild-type mouse kidneys were cultured under different concentrations of pyruvate supplementation for 72 h in Dulbecco's Modified Eagle Medium (Gibco, 41965-039) supplemented with 10% fetal bovine serum (ThermoFisher, 10500064), GlutaMAX (Sigma Aldrich, 35350038) and antibiotics. Cultured embryonic kidneys were analyzed by immunostaining for calbindin (1:500, Santa Cruz Biotechnology, sc-7691), which labels the ureteric bud epithelium, allowing us to examine the effect of pyruvate concentration on ureteric bud branching.

### Hematoxylin-Eosin staining, immunofluorescence detection of nephron progenitors and precursors, imaging and quantification

P1 kidneys from *Pycr1/2* mating were collected and fixed with 4% paraformaldehyde, after which they were processed with paraffin and sectioned according to standard procedures. Hematoxylin-Eosin and immunofluorescence staining, and imaging procedures were performed as previously described (Conaghan et al., 1993). The following primary antibodies were used: anti-cadherin 6 (1:400, a gift from Greg Dressler, University of Michigan, Ann Arbor, MI, USA), anti-calbindin (1:500, Santa Cruz Biotechnology, sc-7691), anti-cleaved caspase 3 (1:200, Cell Signaling Technologies, 9664), anti-cyclin D1 (1:400, PharMingen, 14726E, lot 7823-04), anti-phospho-histone H3 (1:500, Cell Signaling Technologies, 9706), anti-PAX2 (1:400, Invitrogen, 71-6000), anti-TOM20 (1:300, Santa Cruz Biotechnology, sc-19554) and anti-SIX2 (1:500, ProteinTech, 11562-1-AP). Alexa Fluor-conjugated secondary antibodies (1:400; Jackson ImmunoResearch) were used. Images were acquired using a Zeiss AxioImager wide-field microscope with 40× objective and and scanned with a Pannoramic 250 Flash II with 20× objective. Quantification of nephron progenitor cells, ureteric tips and T-buds was performed blindly by three independent researchers each counting 15 medullar sections in two wild-type and two double-knockout *Pycr1/2* kidneys. The means of the three counts were used to calculate the number of cortical nephron progenitors per each ureteric tip.

## Supplementary Material

10.1242/develop.200986_sup1 Supplementary information

## References

[DEV200986C1] Aguilera, O., Munoz-Sagastibelza, M., Torrejon, B., Borrero-Palacios, A., Del Puerto-Nevado, L., Martinez-Useros, J., Rodriguez-Remirez, M., Zazo, S., Garcia, E., Fraga, M. et al. (2016). Vitamin C uncouples the Warburg metabolic switch in KRAS mutant colon cancer. *Oncotarget* 7, 47954-47965. 10.18632/oncotarget.10087 27323830 PMC5216991

[DEV200986C2] Allmeroth, K., Kim, C. S., Annibal, A., Pouikli, A., Koester, J., Derisbourg, M. J., Andrés Chacón-Martínez, C., Latza, C., Antebi, A. and Tessarz, P. (2021). N 1-acetylspermidine is a determinant of hair follicle stem cell fate. *J. Cell Sci.* 134, jcs252767. 10.1242/jcs.252767 33973637 PMC8182411

[DEV200986C3] Anand, S., Khanam, M. A. and Finkelstein, F. O. (2014). Global perspective of kidney disease. In *Nutrition in Kidney Disease* (L. Byham-Gray, J. Burrowes, G. Chertow, eds), pp. 11-23. Springer. 10.1007/978-1-62703-685-6_2

[DEV200986C4] Ashburner, M., Ball, C. A., Blake, J. A., Botstein, D., Butler, H., Cherry, J. M., Davis, A. P., Dolinski, K., Dwight, S. S. and Eppig, J. T. (2000). Gene ontology: tool for the unification of biology. *Nat. Genet.* 25, 25-29. 10.1038/75556 10802651 PMC3037419

[DEV200986C5] Atzler, D., Schwedhelm, E. and Zeller, T. (2014). Integrated genomics and metabolomics in nephrology. *Nephrol. Dial. Transplant.* 29, 1467-1474. 10.1093/ndt/gft492 24366899

[DEV200986C6] Avila, A., Vidal, P. M., Tielens, S., Morelli, G., Laguesse, S., Harvey, R. J., Rigo, J.-M. and Nguyen, L. (2014). Glycine receptors control the generation of projection neurons in the developing cerebral cortex. *Cell Death Differ.* 21, 1696-1708. 10.1038/cdd.2014.75 24926615 PMC4211368

[DEV200986C98] Belanger, L. F., Roy, S., Tremblay, M., Brott, B., Steff, A. M., Mourad, W., Hugo, P., Erikson, R. and Charron, J. (2003). Mek2 is dispensable for mouse growth and development. *Mol. Cell Biol.* 23, 4778-4787. 10.1128/MCB.23.14.4778-4787.200 12832465 PMC162209

[DEV200986C7] Bertram, J. F., Douglas-Denton, R. N., Diouf, B., Hughson, M. D. and Hoy, W. E. (2011). Human nephron number: implications for health and disease. *Pediatr. Nephrol.* 26, 1529-1533. 10.1007/s00467-011-1843-8 21604189

[DEV200986C8] Bindea, G., Mlecnik, B., Hackl, H., Charoentong, P., Tosolini, M., Kirilovsky, A., Fridman, W.-H., Pagès, F., Trajanoski, Z. and Galon, J. (2009). ClueGO: a cytoscape plug-in to decipher functionally grouped gene ontology and pathway annotation networks. *Bioinformatics* 25, 1091-1093. 10.1093/bioinformatics/btp101 19237447 PMC2666812

[DEV200986C9] Bouman, L., Schlierf, A., Lutz, A. K., Shan, J., Deinlein, A., Kast, J., Galehdar, Z., Palmisano, V., Patenge, N., Berg, D. et al. (2011). Parkin is transcriptionally regulated by ATF4: evidence for an interconnection between mitochondrial stress and ER stress. *Cell Death Differ.* 18, 769-782. 10.1038/cdd.2010.142 21113145 PMC3131924

[DEV200986C10] Brown, A. C., Muthukrishnan, S. D., Guay, J. A., Adams, D. C., Schafer, D. A., Fetting, J. L. and Oxburgh, L. (2013). Role for compartmentalization in nephron progenitor differentiation. *Proc. Natl. Acad. Sci. USA* 110, 4640-4645. 10.1073/pnas.1213971110 23487745 PMC3607044

[DEV200986C11] Brown, A. C., Muthukrishnan, S. D. and Oxburgh, L. (2015). A synthetic niche for nephron progenitor cells. *Dev. Cell* 34, 229-241. 10.1016/j.devcel.2015.06.021 26190145 PMC4519427

[DEV200986C12] Brunskill, E. W., Park, J. S., Chung, E., Chen, F., Magella, B. and Potter, S. S. (2014). Single cell dissection of early kidney development: multilineage priming. *Development* 141, 3093-3101. 10.1242/dev.110601 25053437 PMC4197661

[DEV200986C13] Butcher, L., Coates, A., Martin, K. L., Rutherford, A. J. and Leese, H. J. (1998). Metabolism of pyruvate by the early human embryo. *Biol. Reprod.* 58, 1054-1056. 10.1095/biolreprod58.4.1054 9546739

[DEV200986C14] Cargill, K. and Sims-Lucas, S. (2018). Metabolic requirements of the nephron. *Pediatr. Nephrol.* 35, 1-8. 10.1007/s00467-018-4157-2 30554363

[DEV200986C15] Cargill, K., Hemker, S. L., Clugston, A., Murali, A., Mukherjee, E., Liu, J., Bushnell, D., Bodnar, A. J., Saifudeen, Z., Ho, J. et al. (2019). Von Hippel-Lindau acts as a metabolic switch controlling nephron progenitor differentiation. *J. Am. Soc. Nephrol.* 30, 1192-1205. 10.1681/ASN.2018111170 31142573 PMC6622426

[DEV200986C16] Casalino, L., Comes, S., Lambazzi, G., De Stefano, B., Filosa, S., De Falco, S., De Cesare, D., Minchiotti, G. and Patriarca, E. J. (2011). Control of embryonic stem cell metastability by L-proline catabolism. *J. Mol. Cell Biol.* 3, 108-122. 10.1093/jmcb/mjr001 21307025

[DEV200986C17] Cermola, F., D'Aniello, C., Tatè, R., De Cesare, D., Martinez-Arias, A., Minchiotti, G. and Patriarca, E. J. (2021). Gastruloid development competence discriminates different states of pluripotency. *Stem Cell Rep.* 16, 354-369. 10.1016/j.stemcr.2020.12.013 PMC7878839 33482102

[DEV200986C18] Cheng, J., Li, W., Kang, B., Zhou, Y., Song, J., Dan, S., Yang, Y., Zhang, X., Li, J., Yin, S. et al. (2015). Tryptophan derivatives regulate the transcription of Oct4 in stem-like cancer cells. *Nat. Commun.* 6, 7209. 10.1038/ncomms8209 26059097 PMC4490363

[DEV200986C19] Conaghan, J., Handyside, A. H., Winston, R. M. and Leese, H. J. (1993). Effects of pyruvate and glucose on the development of human preimplantation embryos in vitro. *J. Reprod. Fertil.* 99, 87-95. 10.1530/jrf.0.0990087 8283458

[DEV200986C20] Costantini, F. and Kopan, R. (2010). Patterning a complex organ: branching morphogenesis and nephron segmentation in kidney development. *Dev. Cell* 18, 698-712. 10.1016/j.devcel.2010.04.008 20493806 PMC2883254

[DEV200986C21] D'Aniello, C., Habibi, E., Cermola, F., Paris, D., Russo, F., Fiorenzano, A., Di Napoli, G., Melck, D. J., Cobellis, G., Angelini, C. et al. (2017). Vitamin C and l-proline antagonistic effects capture alternative states in the pluripotency continuum. *Stem Cell Rep.* 8, 1-10. 10.1016/j.stemcr.2016.11.011 PMC5233408 28017658

[DEV200986C22] Davidson, A. J., Lewis, P., Przepiorski, A. and Sander, V. (2019). Turning mesoderm into kidney. *Semin. Cell Dev. Biol.* 91, 86-93. 10.1016/j.semcdb.2018.08.016 30172050

[DEV200986C23] De Ingeniis, J., Ratnikov, B., Richardson, A. D., Scott, D. A., Aza-Blanc, P., De, S. K., Kazanov, M., Pellecchia, M., Ronai, Z., Osterman, A. L. et al. (2012). Functional specialization in proline biosynthesis of melanoma. *PLoS One* 7, e45190. 10.1371/journal.pone.0045190 23024808 PMC3443215

[DEV200986C24] de Koning, T. J. (2017). Amino acid synthesis deficiencies. *J. Inherit. Metab. Dis.* 40, 609-620. 10.1007/s10545-017-0063-1 28653176 PMC5500668

[DEV200986C25] Escande-Beillard, N., Loh, A., Saleem, S. N., Kanata, K., Hashimoto, Y., Altunoglu, U., Metoska, A., Grandjean, J., Ng, F. M., Pomp, O. et al. (2020). Loss of PYCR2 causes neurodegeneration by increasing cerebral glycine levels via SHMT2. *Neuron* 107, 82-94.e6. 10.1016/j.neuron.2020.03.028 32330411

[DEV200986C26] Fiehn, O. (2002). Metabolomics--the link between genotypes and phenotypes. *Plant Mol. Biol.* 48, 155-171. 10.1023/A:1013713905833 11860207

[DEV200986C27] Fisher, C. E., Michael, L., Barnett, M. W. and Davies, J. A. (2001). Erk MAP kinase regulates branching morphogenesis in the developing mouse kidney. *Development* 128, 4329-4338. 10.1242/dev.128.21.4329 11684667

[DEV200986C28] Grimley, E. and Dressler, G. R. (2018). Are Pax proteins potential therapeutic targets in kidney disease and cancer? *Kidney Int.* 94, 259-267. 10.1016/j.kint.2018.01.025 29685496 PMC6054895

[DEV200986C29] Guijas, C., Montenegro-Burke, J. R., Domingo-Almenara, X., Palermo, A., Warth, B., Hermann, G., Koellensperger, G., Huan, T., Uritboonthai, W., Aisporna, A. E. et al. (2018). METLIN: a technology platform for identifying knowns and unknowns. *Anal. Chem.* 90, 3156-3164. 10.1021/acs.analchem.7b04424 29381867 PMC5933435

[DEV200986C30] Gupta, V. and Bamezai, R. N. (2010). Human pyruvate kinase M2: a multifunctional protein. *Protein Sci.* 19, 2031-2044. 10.1002/pro.505 20857498 PMC3005776

[DEV200986C31] Hartman, H. A., Lai, H. L. and Patterson, L. T. (2007). Cessation of renal morphogenesis in mice. *Dev. Biol.* 310, 379-387. 10.1016/j.ydbio.2007.08.021 17826763 PMC2075093

[DEV200986C32] Hida, M., Omori, S. and Awazu, M. (2002). ERK and p38 MAP kinase are required for rat renal development. *Kidney Int.* 61, 1252-1262. 10.1046/j.1523-1755.2002.00273.x 11918731

[DEV200986C33] Hinchliffe, S. A., Sargent, P. H., Howard, C. V., Chan, Y. F. and van Velzen, D. (1991). Human intrauterine renal growth expressed in absolute number of glomeruli assessed by the disector method and Cavalieri principle. *Lab. Invest.* 64, 777-784. 2046329

[DEV200986C34] Ihermann-Hella, A. and Kuure, S. (2019). Mouse ex vivo kidney culture methods. *Methods Mol. Biol.* 1926, 23-30. 10.1007/978-1-4939-9021-4_2 30742259

[DEV200986C35] Ihermann-Hella, A., Lume, M., Miinalainen, I. J., Pirttiniemi, A., Gui, Y., Peränen, J., Charron, J., Saarma, M., Costantini, F. and Kuure, S. (2014). Mitogen-activated protein kinase (MAPK) pathway regulates branching by remodeling epithelial cell adhesion. *PLoS Genet.* 10, e1004193. 10.1371/journal.pgen.1004193 24603431 PMC3945187

[DEV200986C36] Ihermann-Hella, A., Hirashima, T., Kupari, J., Kurtzeborn, K., Li, H., Kwon, H. N., Cebrian, C., Soofi, A., Dapkunas, A., Miinalainen, I. et al. (2018). Dynamic MAPK/ERK activity sustains nephron progenitors through niche regulation and primes precursors for differentiation. *Stem Cell Rep.* 11, 912-928. 10.1016/j.stemcr.2018.08.012 PMC6178244 30220628

[DEV200986C37] Jain, S. and Chen, F. (2019). Developmental pathology of congenital kidney and urinary tract anomalies. *Clin. Kidney J.* 12, 382-399. 10.1093/ckj/sfy112 31198539 PMC6543978

[DEV200986C38] Jones, M. E. (1985). Conversion of glutamate to ornithine and proline: pyrroline-5-carboxylate, a possible modulator of arginine requirements. *J. Nutr.* 115, 509-515. 10.1093/jn/115.4.509 2858518

[DEV200986C39] Karner, C. M., Das, A., Ma, Z., Self, M., Chen, C., Lum, L., Oliver, G. and Carroll, T. J. (2011). Canonical Wnt9b signaling balances progenitor cell expansion and differentiation during kidney development. *Development* 138, 1247-1257. 10.1242/dev.057646 21350016 PMC3050658

[DEV200986C40] Kim, C. S., Ding, X., Allmeroth, K., Biggs, L. C., Kolenc, O. I., L'Hoest, N., Chacon-Martinez, C. A., Edlich-Muth, C., Giavalisco, P., Quinn, K. P. et al. (2020). Glutamine metabolism controls stem cell fate reversibility and long-term maintenance in the hair follicle. *Cell Metab.* 32, 629-642.e8. 10.1016/j.cmet.2020.08.011 32905798

[DEV200986C41] Kimura, T., Takeda, S., Sagiya, Y., Gotoh, M., Nakamura, Y. and Arakawa, H. (2003). Impaired function of p53R2 in Rrm2b-null mice causes severe renal failure through attenuation of dNTP pools. *Nat. Genet.* 34, 440-445. 10.1038/ng1212 12858174

[DEV200986C42] Kitano, H. (2002). Systems biology: a brief overview. *Science* 295, 1662-1664. 10.1126/science.1069492 11872829

[DEV200986C43] Kobayashi, A., Valerius, M. T., Mugford, J. W., Carroll, T. J., Self, M., Oliver, G. and McMahon, A. P. (2008). Six2 defines and regulates a multipotent self-renewing nephron progenitor population throughout mammalian kidney development. *Cell Stem Cell* 3, 169-181. 10.1016/j.stem.2008.05.020 18682239 PMC2561900

[DEV200986C44] Krafczyk, R., Qi, F., Sieber, A., Mehler, J., Jung, K., Frishman, D. and Lassak, J. (2021). Proline codon pair selection determines ribosome pausing strength and translation efficiency in bacteria. *Commun. Biol.* 4, 589. 10.1038/s42003-021-02115-z 34002016 PMC8129111

[DEV200986C45] Kuo, M. L., Lee, M. B., Tang, M., den Besten, W., Hu, S., Sweredoski, M. J., Hess, S., Chou, C. M., Changou, C. A., Su, M. et al. (2016). PYCR1 and PYCR2 interact and collaborate with RRM2B to protect cells from overt oxidative stress. *Sci. Rep.* 6, 18846. 10.1038/srep18846 26733354 PMC4702135

[DEV200986C46] Kurtzeborn, K., Kwon, H. N. and Kuure, S. (2019). MAPK/ERK signaling in regulation of renal differentiation. *Int. J. Mol. Sci.* 20, 1779. 10.3390/ijms20071779 PMC6479953 30974877

[DEV200986C47] Kurtzeborn, K., Kwon, H. N., Iaroshenko, V., Faisal, I., Ambroz, M., Jin, X., Qureshi, T., Kupari, J., Ihermann-Hella, A., Vaananen, J. et al. (2022). Comparative whole-genome transcriptome analysis in renal cell populations reveals high tissue specificity of MAPK/ERK targets in embryonic kidney. *BMC Biol.* 20, 112. 10.1186/s12915-022-01309-z 35550069 PMC9102746

[DEV200986C48] Li, Y., Liu, J., Li, W., Brown, A., Baddoo, M., Li, M., Carroll, T., Oxburgh, L., Feng, Y. and Saifudeen, Z. (2015). p53 Enables metabolic fitness and self-renewal of nephron progenitor cells. *Development* 142, 1228-1241. 10.1242/dev.111617 25804735 PMC4378244

[DEV200986C49] Li, Z., Araoka, T., Wu, J., Liao, H. K., Li, M., Lazo, M., Zhou, B., Sui, Y., Wu, M. Z., Tamura, I. et al. (2016). 3D culture supports long-term expansion of mouse and human nephrogenic progenitors. *Cell Stem Cell* 19, 516-529. 10.1016/j.stem.2016.07.016 27570066 PMC7832086

[DEV200986C50] Li, H., Hohenstein, P. and Kuure, S. (2021a). Embryonic kidney development, stem cells and the origin of Wilms tumor. *Genes* 12, 318. 10.3390/genes12020318 33672414 PMC7926385

[DEV200986C51] Li, H., Kurtzeborn, K., Kupari, J., Gui, Y., Siefker, E., Lu, B., Matlik, K., Olfat, S., Montano-Rodriguez, A. R., Huh, S. H. et al. (2021b). Postnatal prolongation of mammalian nephrogenesis by excess fetal GDNF. *Development* 148, dev197475. 10.1242/dev.197475 34032268 PMC8180252

[DEV200986C52] Lindström, N. O., Carragher, N. O. and Hohenstein, P. (2015). The PI3K pathway balances self-renewal and differentiation of nephron progenitor cells through β-catenin signaling. *Stem Cell Rep.* 4, 551-560. 10.1016/j.stemcr.2015.01.021 PMC4400645 25754203

[DEV200986C53] Liu, J., Edgington-Giordano, F., Dugas, C., Abrams, A., Katakam, P., Satou, R. and Saifudeen, Z. (2017). Regulation of nephron progenitor cell self-renewal by intermediary metabolism. *J. Am. Soc. Nephrol.* 28, 3323-3335. 10.1681/ASN.2016111246 28754792 PMC5661282

[DEV200986C54] Liu, N., Dai, Z., Zhang, Y., Chen, J., Yang, Y., Wu, G., Tso, P. and Wu, Z. (2019). Maternal L-proline supplementation enhances fetal survival, placental development, and nutrient transport in mice. *Biol. Reprod.* 100, 1073-1081. 10.1093/biolre/ioy240 30418498 PMC6698749

[DEV200986C55] Luyckx, V. A. and Brenner, B. M. (2010). The clinical importance of nephron mass. *J. Am. Soc. Nephrol.* 21, 898-910. 10.1681/ASN.2009121248 20150537

[DEV200986C56] Luyckx, V. A., Tonelli, M. and Stanifer, J. W. (2018). The global burden of kidney disease and the sustainable development goals. *Bull. W.H.O* 96, 414. 10.2471/BLT.17.206441 29904224 PMC5996218

[DEV200986C57] Maejima, Y., Galeotti, J., Molkentin, J. D., Sadoshima, J. and Zhai, P. (2012). Constitutively active MEK1 rescues cardiac dysfunction caused by overexpressed GSK-3α during aging and hemodynamic pressure overload. *Am. J. Physiol. Heart Circ. Physiol.* 303, H979-H988. 10.1152/ajpheart.00415.2012 22904158 PMC3774208

[DEV200986C58] Magadum, A., Singh, N., Kurian, A. A., Munir, I., Mehmood, T., Brown, K., Sharkar, M. T. K., Chepurko, E., Sassi, Y., Oh, J. G. et al. (2020). Pkm2 regulates cardiomyocyte cell cycle and promotes cardiac regeneration. *Circulation* 141, 1249-1265. 10.1161/CIRCULATIONAHA.119.043067 32078387 PMC7241614

[DEV200986C59] Makayes, Y., Resnick, E., Hinden, L., Aizenshtein, E., Shlomi, T., Kopan, R., Nechama, M. and Volovelsky, O. (2021). Increasing mTORC1 pathway activity or methionine supplementation during pregnancy reverses the negative effect of maternal malnutrition on the developing kidney. *J. Am. Soc. Nephrol.* 32, 1898-1912. 10.1681/ASN.2020091321 33958489 PMC8455268

[DEV200986C60] McKillop, A. M. and Flatt, P. R. (2011). Emerging applications of metabolomic and genomic profiling in diabetic clinical medicine. *Diabetes Care* 34, 2624-2630. 10.2337/dc11-0837 22110171 PMC3220869

[DEV200986C61] Melnikov, S., Mailliot, J., Rigger, L., Neuner, S., Shin, B. S., Yusupova, G., Dever, T. E., Micura, R. and Yusupov, M. (2016). Molecular insights into protein synthesis with proline residues. *EMBO Rep.* 17, 1776-1784. 10.15252/embr.201642943 27827794 PMC5283605

[DEV200986C62] Moussaieff, A., Rouleau, M., Kitsberg, D., Cohen, M., Levy, G., Barasch, D., Nemirovski, A., Shen-Orr, S., Laevsky, I., Amit, M. et al. (2015). Glycolysis-mediated changes in acetyl-CoA and histone acetylation control the early differentiation of embryonic stem cells. *Cell Metab.* 21, 392-402. 10.1016/j.cmet.2015.02.002 25738455

[DEV200986C63] Murugapoopathy, V. and Gupta, I. R. (2020). A primer on congenital anomalies of the kidneys and urinary tracts (CAKUT). *Clin. J. Am. Soc. Nephrol.* 15, 723-731. 10.2215/CJN.12581019 32188635 PMC7269211

[DEV200986C64] Naiman, N., Fujioka, K., Fujino, M., Valerius, M. T., Potter, S. S., McMahon, A. P. and Kobayashi, A. (2017). Repression of interstitial identity in nephron progenitor cells by Pax2 establishes the nephron-interstitium boundary during kidney development. *Dev. Cell* 41, 349-365.e3. 10.1016/j.devcel.2017.04.022 28535371 PMC5532731

[DEV200986C65] Nam, H., Chung, B. C., Kim, Y., Lee, K. and Lee, D. (2009). Combining tissue transcriptomics and urine metabolomics for breast cancer biomarker identification. *Bioinformatics* 25, 3151-3157. 10.1093/bioinformatics/btp558 19783829

[DEV200986C66] O'Brien, L. L. (2019). Nephron progenitor cell commitment: striking the right balance. *Semin. Cell Dev. Biol.* 91, 94-103. 10.1016/j.semcdb.2018.07.017 30030141

[DEV200986C67] Ogata, H., Goto, S., Sato, K., Fujibuchi, W., Bono, H. and Kanehisa, M. (1999). KEGG: Kyoto Encyclopedia of genes and genomes. *Nucleic Acids Res.* 27, 29-34. 10.1093/nar/27.1.29 9847135 PMC148090

[DEV200986C68] Oxburgh, L. (2018). Kidney nephron determination. *Annu. Rev. Cell Dev. Biol.* 34, 427-450. 10.1146/annurev-cellbio-100616-060647 30125139

[DEV200986C69] Pang, H., Jia, W. and Hu, Z. (2019). Emerging applications of metabolomics in clinical pharmacology. *Clin. Pharmacol. Ther.* 106, 544-556. 10.1002/cpt.1538 31173340

[DEV200986C70] Patriarca, E. J., Cermola, F., D'Aniello, C., Fico, A., Guardiola, O., De Cesare, D. and Minchiotti, G. (2021). The multifaceted roles of proline in cell behavior. *Front. Cell Dev. Biol.* 9, 728576. 10.3389/fcell.2021.728576 34458276 PMC8397452

[DEV200986C71] Powell, D. R., Desai, U., Sparks, M. J., Hansen, G., Gay, J., Schrick, J., Shi, Z. Z., Hicks, J. and Vogel, P. (2005). Rapid development of glomerular injury and renal failure in mice lacking p53R2. *Pediatr. Nephrol.* 20, 432-440. 10.1007/s00467-004-1696-5 15723268

[DEV200986C72] Purcell, N. H., Wilkins, B. J., York, A., Saba-El-Leil, M. K., Meloche, S., Robbins, J. and Molkentin, J. D. (2007). Genetic inhibition of cardiac ERK1/2 promotes stress-induced apoptosis and heart failure but has no effect on hypertrophy in vivo. *Proc. Natl. Acad. Sci. USA* 104, 14074-14079. 10.1073/pnas.0610906104 17709754 PMC1955824

[DEV200986C73] Reversade, B., Escande-Beillard, N., Dimopoulou, A., Fischer, B., Chng, S. C., Li, Y., Shboul, M., Tham, P. Y., Kayserili, H., Al-Gazali, L. et al. (2009). Mutations in PYCR1 cause cutis laxa with progeroid features. *Nat. Genet.* 41, 1016-1021. 10.1038/ng.413 19648921

[DEV200986C74] Rossi, R., Dalle-Donne, I., Milzani, A. and Giustarini, D. (2006). Oxidized forms of glutathione in peripheral blood as biomarkers of oxidative stress. *Clin. Chem.* 52, 1406-1414. 10.1373/clinchem.2006.067793 16690733

[DEV200986C75] Rumballe, B. A., Georgas, K. M., Combes, A. N., Ju, A. L., Gilbert, T. and Little, M. H. (2011). Nephron formation adopts a novel spatial topology at cessation of nephrogenesis. *Dev. Biol.* 360, 110-122. 10.1016/j.ydbio.2011.09.011 21963425 PMC6186757

[DEV200986C76] Ryan, D., Sutherland, M. R., Flores, T. J., Kent, A. L., Dahlstrom, J. E., Puelles, V. G., Bertram, J. F., McMahon, A. P., Little, M. H., Moore, L. et al. (2018). Development of the human fetal kidney from mid to late gestation in male and female infants. *EBioMedicine* 27, 275-283. 10.1016/j.ebiom.2017.12.016 29329932 PMC5828465

[DEV200986C99] Scholl, F. A., Dumesic, P. A., Barragan, D. I., Harada, K., Bissonauth, V., Charron, J. and Khavari, P. A. (2007). Mek1/2 MAPK kinases are essential for Mammalian development, homeostasis, and Raf-induced hyperplasia. *Dev. Cell* 12, 615-629. 10.1016/j.devcel.2007.03.009 17419998

[DEV200986C77] Schrader, M. and Fahimi, H. D. (2006). Peroxisomes and oxidative stress. *Biochim. Biophys. Acta* 1763, 1755-1766. 10.1016/j.bbamcr.2006.09.006 17034877

[DEV200986C78] Self, M., Lagutin, O. V., Bowling, B., Hendrix, J., Cai, Y., Dressler, G. R. and Oliver, G. (2006). Six2 is required for suppression of nephrogenesis and progenitor renewal in the developing kidney. *EMBO J.* 25, 5214-5228. 10.1038/sj.emboj.7601381 17036046 PMC1630416

[DEV200986C79] Shannon, P., Markiel, A., Ozier, O., Baliga, N. S., Wang, J. T., Ramage, D., Amin, N., Schwikowski, B. and Ideker, T. (2003). Cytoscape: a software environment for integrated models of biomolecular interaction networks. *Genome Res.* 13, 2498-2504. 10.1101/gr.1239303 14597658 PMC403769

[DEV200986C80] Sharma, K., Karl, B., Mathew, A. V., Gangoiti, J. A., Wassel, C. L., Saito, R., Pu, M., Sharma, S., You, Y. H., Wang, L. et al. (2013). Metabolomics reveals signature of mitochondrial dysfunction in diabetic kidney disease. *J. Am. Soc. Nephrol.* 24, 1901-1912. 10.1681/ASN.2013020126 23949796 PMC3810086

[DEV200986C81] Shyh-Chang, N. and Ng, H. H. (2017). The metabolic programming of stem cells. *Genes Dev.* 31, 336-346. 10.1101/gad.293167.116 28314766 PMC5358754

[DEV200986C82] Someya, S., Tohyama, S., Kameda, K., Tanosaki, S., Morita, Y., Sasaki, K., Kang, M. I., Kishino, Y., Okada, M., Tani, H. et al. (2021). Tryptophan metabolism regulates proliferative capacity of human pluripotent stem cells. *iScience* 24, 102090. 10.1016/j.isci.2021.102090 33615198 PMC7878994

[DEV200986C83] Stum, M. G., Tadenev, A. L. D., Seburn, K. L., Miers, K. E., Poon, P. P., McMaster, C. R., Robinson, C., Kane, C., Silva, K. A., Cliften, P. F. et al. (2021). Genetic analysis of Pycr1 and Pycr2 in mice. *Genetics* 218, iyab048. 33734376 10.1093/genetics/iyab048 PMC8128379

[DEV200986C84] Tchantchou, F., Miller, C., Goodfellow, M., Puche, A. and Fiskum, G. (2021). Hypobaria-induced oxidative stress facilitates homocysteine transsulfuration and promotes glutathione oxidation in rats with mild traumatic brain injury. *J. Cent. Nerv. Syst. Dis.* 13, 1179573520988193. 10.1177/1179573520988193 33597815 PMC7863175

[DEV200986C85] Tortelote, G. G., Colón-Leyva, M. and Saifudeen, Z. (2021). Metabolic programming of nephron progenitor cell fate. *Pediatr. Nephrol.* 36, 2155-2164. 10.1007/s00467-020-04752-8 33089379 PMC10734399

[DEV200986C86] Tran, M. T., Zsengeller, Z. K., Berg, A. H., Khankin, E. V., Bhasin, M. K., Kim, W., Clish, C. B., Stillman, I. E., Karumanchi, S. A., Rhee, E. P. et al. (2016). PGC1α drives NAD biosynthesis linking oxidative metabolism to renal protection. *Nature* 531, 528-532. 10.1038/nature17184 26982719 PMC4909121

[DEV200986C87] Volovelsky, O., Nguyen, T., Jarmas, A. E., Combes, A. N., Wilson, S. B., Little, M. H., Witte, D. P., Brunskill, E. W. and Kopan, R. (2018). Hamartin regulates cessation of mouse nephrogenesis independently of Mtor. *Proc. Natl. Acad. Sci. USA* 115, 5998-6003. 10.1073/pnas.1712955115 29784808 PMC6003359

[DEV200986C88] Washington, J. M., Rathjen, J., Felquer, F., Lonic, A., Bettess, M. D., Hamra, N., Semendric, L., Tan, B. S., Lake, J. A., Keough, R. A. et al. (2010). L-Proline induces differentiation of ES cells: a novel role for an amino acid in the regulation of pluripotent cells in culture. *Am. J. Physiol. Cell Physiol.* 298, C982-C992. 10.1152/ajpcell.00498.2009 20164384

[DEV200986C89] Weckwerth, W. (2003). Metabolomics in systems biology. *Annu. Rev. Plant Biol.* 54, 669-689. 10.1146/annurev.arplant.54.031902.135014 14503007

[DEV200986C90] Wishart, D. S., Feunang, Y. D., Marcu, A., Guo, A. C., Liang, K., Vazquez-Fresno, R., Sajed, T., Johnson, D., Li, C., Karu, N. et al. (2018). HMDB 4.0: the human metabolome database for 2018. *Nucleic Acids Res.* 46, D608-D617. 10.1093/nar/gkx1089 29140435 PMC5753273

[DEV200986C91] Wu, H., Li, Z., Yang, P., Zhang, L., Fan, Y. and Li, Z. (2014). PKM2 depletion induces the compensation of glutaminolysis through β-catenin/c-Myc pathway in tumor cells. *Cell. Signal.* 26, 2397-2405. 10.1016/j.cellsig.2014.07.024 25041845

[DEV200986C92] Xia, J. and Wishart, D. S. (2010). MSEA: a web-based tool to identify biologically meaningful patterns in quantitative metabolomic data. *Nucleic Acids Res.* 38, W71-W77. 10.1093/nar/gkq329 20457745 PMC2896187

[DEV200986C93] Yang, W., Zheng, Y., Xia, Y., Ji, H., Chen, X., Guo, F., Lyssiotis, C. A., Aldape, K., Cantley, L. C. and Lu, Z. (2012). ERK1/2-dependent phosphorylation and nuclear translocation of PKM2 promotes the Warburg effect. *Nat. Cell Biol.* 14, 1295-1304. 10.1038/ncb2629 23178880 PMC3511602

[DEV200986C94] Yang, C., Ko, B., Hensley, C. T., Jiang, L., Wasti, A. T., Kim, J., Sudderth, J., Calvaruso, M. A., Lumata, L., Mitsche, M. et al. (2014). Glutamine oxidation maintains the TCA cycle and cell survival during impaired mitochondrial pyruvate transport. *Mol. Cell* 56, 414-424. 10.1016/j.molcel.2014.09.025 25458842 PMC4268166

[DEV200986C95] Zhang, H., Bagherie-Lachidan, M., Badouel, C., Enderle, L., Peidis, P., Bremner, R., Kuure, S., Jain, S. and McNeill, H. (2019). FAT4 fine-tunes kidney development by regulating RET signaling. *Dev. Cell* 48, 780-792.e4. 10.1016/j.devcel.2019.02.004 30853441 PMC6766079

[DEV200986C96] Zhao, T., Goh, K. J., Ng, H. H. and Vardy, L. A. (2012). A role for polyamine regulators in ESC self-renewal. *Cell Cycle* 11, 4517-4523. 10.4161/cc.22772 23165208 PMC3562295

[DEV200986C97] Zhou, H.-L., Zhang, R., Anand, P., Stomberski, C. T., Qian, Z., Hausladen, A., Wang, L., Rhee, E. P., Parikh, S. M., Karumanchi, S. A. et al. (2019). Metabolic reprogramming by the S-nitroso-CoA reductase system protects against kidney injury. *Nature* 565, 96-100. 10.1038/s41586-018-0749-z 30487609 PMC6318002

