# Supplementary material for: Omics profiling identifies the regulatory functions of the MAPK/ERK pathway in nephron progenitor metabolism
Source: Development. 2022 Oct 3;149(19):dev200986. doi: 10.1242/dev.200986 (PMC9641663; doi:10.1242/dev.200986)
Supplement: Supplementary information [file develop-149-200986-s1.pdf]

Figure 1.

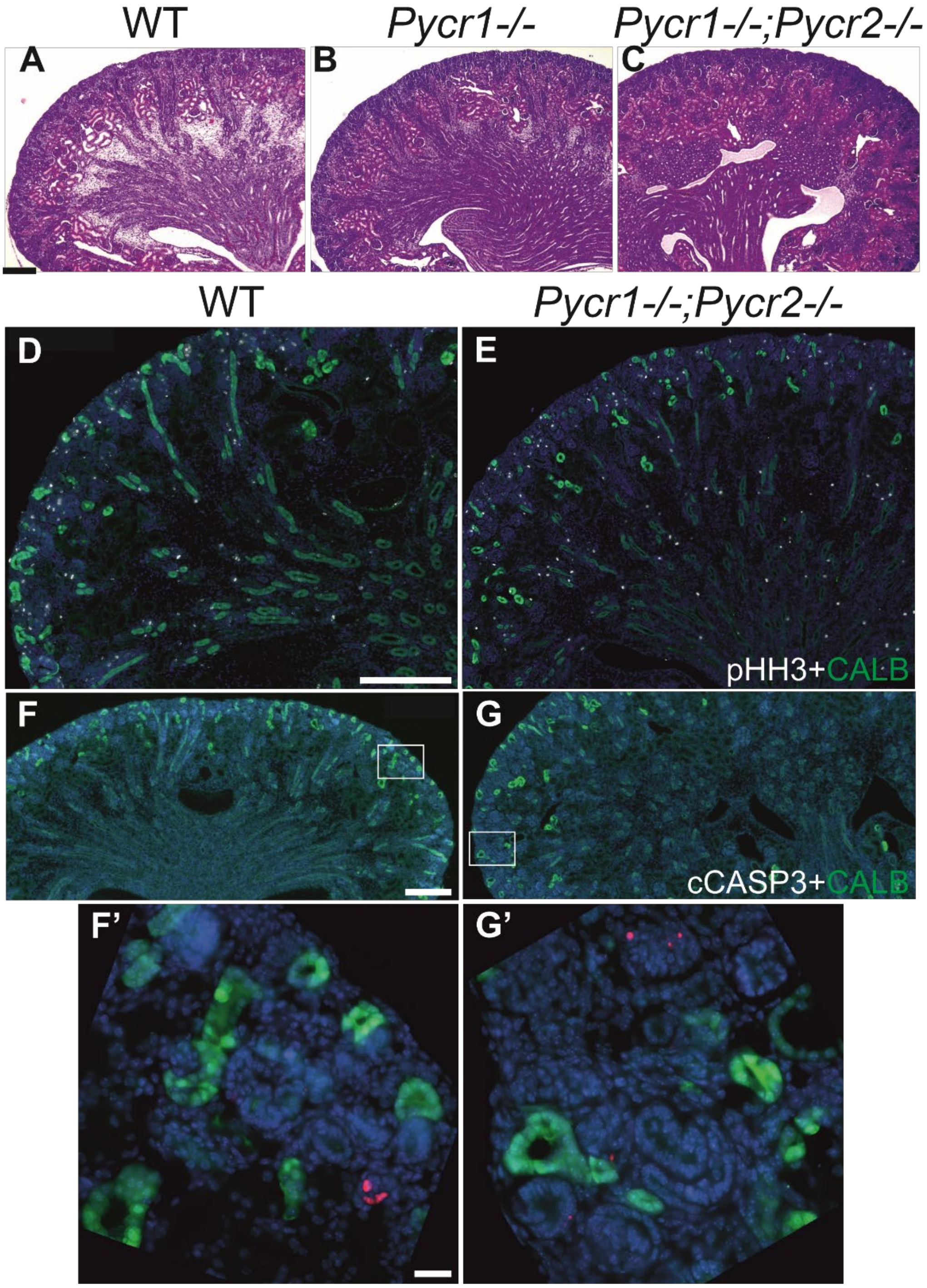

Fig. S1. ***Pycr1*** **and** ***-2*** **knockout kidney characterization.** HE-staining of (**A**) wild type, (**B**) *Pycr1* and (**C**) *Pycr1*$^{-/-}$;*Pycr2*$^{-/-}$ kidneys at postnatal day 1 (P1). Representative examples of proliferation analyses in (**D**) wild type (WT) and (**E**) *Pycr1*$^{-/-}$;*Pycr2*$^{-/-}$ kidneys at P1 by phospho-histone H3 (white) staining. Representative examples of apoptosis detection in (**F**) wild type (WT) and (**G**) *Pycr1*$^{-/-}$;*Pycr2*$^{-/-}$ kidneys at P1 by cleaved-Caspase 3 (white) staining. CALBINDIN (green) visualizes ureteric bud epithelium in D-G'. Squared areas indicated in F and G are shown with higher magnification in (F') and (G'). Scale bar: **A-G**; 200µm, F'-G'; 20µm.

Development • Supplementary information

Figure 2.

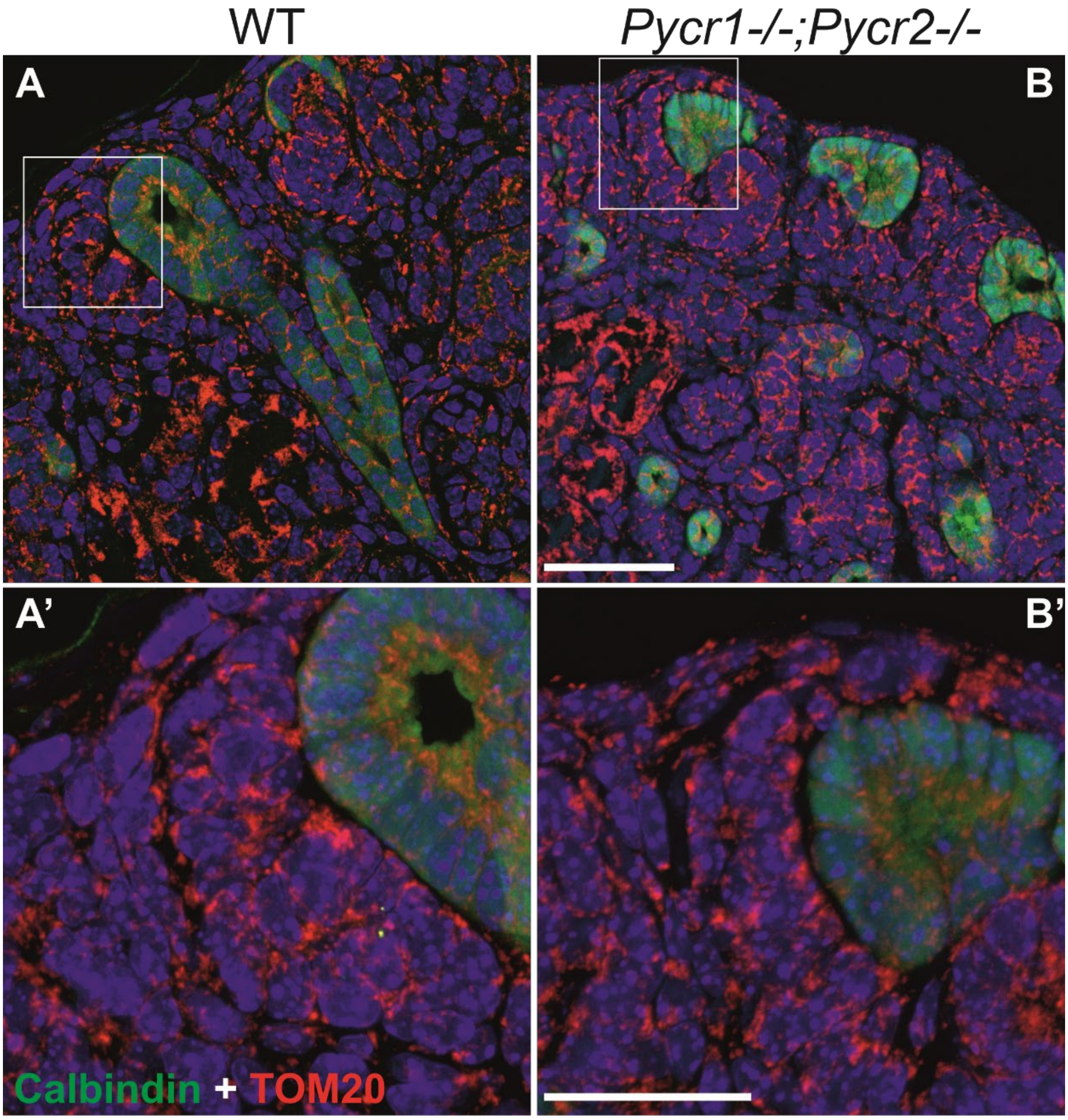

Fig. S2. Mitochondrial morphology in nephron progenitors of *Pycr1* and *-2* knockout kidney. Representative examples of mitochondrial morphology and localization in (**A**) wild type (WT) and (**B**) $Pycr1^{-/-}$;$Pycr2^{-/-}$ kidneys at P1 as visualized by TOM20 (red) staining. CALBINDIN (green) visualizes ureteric bud epithelium. Scale bar: A-B; 50µm, A'-B'; 25µm

Table S1. List of differentially expressed genes

Click here to download Table S1

Table S2. Metabolite marker identification

Click here to download Table S2

Table S3. Metabolic set enrichment analysis (MSEA) of identified metabolites

Click here to download Table S3

Table S4. The correlations between identified metabolites and differentially expressed genes

Click here to download Table S4

Table S5. Comparison of our transcriptional changes to scRNA-seq by Brunskill et al 2014

Click here to download Table S5

Table S6. Analysis of MAPK/ERK target and oxidative stress related gene expressions in MAPK/ERK-deficient nephron progenitors

Click here to download Table S6

Table S7. The metabolite inclusion list of parallel reaction methods

Click here to download Table S7
