# Supplementary material for: Omics profiling identifies the regulatory functions of the MAPK/ERK pathway in nephron progenitor metabolism
Source: Development. 2022 Oct 3;149(19):dev200986. doi: 10.1242/dev.200986 (PMC9641663; doi:10.1242/dev.200986)
Supplement: Supplementary file 1 [file develop-149-200986_review_history.pdf]

Omics profiling identifies the regulatory functions of the MAPK/ ERK pathway in nephron progenitor metabolism

Hyuk Nam Kwon, Kristen Kurtzeborn, Vladislav Iaroshenko, Xing Jin, Abigail Loh, Nathalie Escande-Beillard, Bruno Reversade, Sunghyouk Park and Satu Kuure
DOI: 10.1242/dev.200986

Editor: Irene Miguel-Aliaga

Review timeline

Submission to Review Commons:	18 November 2021
Submission to Development:	25 May 2022
Editorial decision:	18 July 2022
First revision received:	3 August 2022
Accepted:	25 August 2022

Reviewer 1

Evidence, reproducibility and clarity

Summary

In this manuscript Kwon et al use a multi-omics-based approach to identify the MAPK/ERK pathway as contributor to the maintenance and propagation of the developing kidney. This claim is first supported by transcriptional profiling of nephron progenitor cells with conditional inactivation of both Mek genes (Mek1/Mek2) which identifies >5000 differentially expressed genes with further analysis of these genes via RNA-Seq showing mitochondrial metabolism is most effected by MAPK/ERK inactivation. The physiological consequences are then examined via pyruvate titration experiments using E11.5 mouse embryonic kidneys which claimed to show reduced branching of the ureteric buds and double Pycr1 and Pycr2 knockouts revealing defects in NP as evidence by reduced PAX6 and SIX2 staining.

Major Comments:

-The transcriptomic profiling data and further figures identifying the MAPK/ERK pathway is contributing to the maintenance and propagation of nephron progenitor cells. However experiments showing physiological consequences need more detailed analysis. What could, for example, be done is using direct inhibition of MAPK/ERK via UO126 used in previous figures (Fig 2) to check for any morphological consequences on ureteric bud branching in embryonic kidney cultures and to compare against simple pyruvate titration experiments.

-Why is LC/MS analysis done on an embryonic kidney mesenchyme cell line and not on NPs isolated from E13.5 kidneys used for the RNA-seq analysis?

-Further comments on the morphological differences of proline depletion (Pycr1/2 dko) would also be valuable. There seems to be an inversion of expected phenotype particularly in images stained with SIX2. Maybe consider that lack of proline reduces NPC proliferation causing premature differentiation into a renal vesicle. Here, the paper could benefit from specific immunostainings against CDH6 or other epithelial markers.

© 2022. Published by The Company of Biologists under the terms of the Creative Commons Attribution License (https://creativecommons.org/licenses/by/4.0/).

-Is there a potential connection between pyruvate and proline metabolism? If yes, please discuss.

Minor Comments

•Images from figure 6a and 6b showing physiological consequences of pyruvate concentration on embryonic kidneys do not have appropriate scale bars

•Would be good to have statistical analysis for figure 6c to show if the differences between WT and dko lines are statistically significant.

•Would be good to have the information about sample/number of replicates on the graph.

Significance

In summary the manuscript offers a clear and rational identification of the MAPK/ERK pathways as an important mechanism in the maintenance and propagation of nephron progenitor cells. As little is known about the metabolic requirements in renal development, the findings are valuable to the field. However, conclusions regarding the effect of this pathway on morphology of ureteric bud tips and NPC population localisation need further substantiation.

Reviewer 2

Evidence, reproducibility and clarity

Summary:

In the manuscript by Kwon et al a deeper understanding of metabolic changes associated with nephron progenitor differentiation is achieved through multi-disciplinary analysis. Mass spectrometry of a cell line representing normal nephron progenitor behavior in comparison with RNA-seq data brought the researchers to focus specifically on the MAPK/ERK pathway as one of interest in determining the pluripotent or differentiated status of NPs. They tested the effects of MAPK/ERK deficiency on the downstream process of proline metabolism and specifically the genes PYCR1/2 in vivo, both with organ culture and a knockout mouse line. While these in vivo studies did not show a gross phenotype associated with defective embryonic development, the authors conclude that a tightly controlled level of pyruvate and PYCR1/2 activity is necessary to prevent premature differentiation of nephron progenitors.

Criticisms:

1.Overall, this reviewer agrees that the conclusions drawn, while speculative, are factually correct based on the experimentation. However, the experiments performed (especially in vivo) fall short of determining any major evidence on the metabolism of nephron progenitors, and thus this reviewer does not believe that the preliminary findings are significant enough to progress the field.

2.The metabolomics data and RNA seq data (while not specifically provided in this manuscript, but appears to be in preparation for a separate manuscript) are no doubt solid and interesting, they fail to be properly translated into cellular behavior or mechanism.

3.The lack of a phenotype in the knockout mouse leaves the reader unconvinced of how this pathway is a "gatekeeper". We suggest that the paper be supported with significantly more in vivo experiments including various time points and more defined read-outs of metabolic activity.

4.The LC/MS was performed on a cell line, rather than in vivo, which clouds the results especially since the specific cell line is never characterized in terms of timing of development, genetic changes such a immortalization etc.

5.Figure 4 is difficult to understand as it seems unfocused, underlining a lack of specific direction in the study of metabolics

© 2022. Published by The Company of Biologists under the terms of the Creative Commons Attribution License (https://creativecommons.org/licenses/by/4.0/).

6.We strongly suggest that different time points be tested in a single experiment to prove a defect rather than a delay

7.Additional markers of NP differentiation and increased number of samples quantified in Figure 6 would strengthen the results

8.Neither scale bars or statistical testing were shown in Figure 6 and the images in Figure 6B are shown at inconsistent magnifications

9.There are several areas of the text that need adequate proofreading including typos and syntax. It is very clear that several people contributed to the manuscript leaving the reader with a very piecemeal combination of authors voices.

Significance

This reviewer does not see any technical errors in experimentation, simply over-interpretation of significance of the findings. While this manuscript provides an interesting factoid about PYCR1/2, it is not enough to advance the field in either cellular/genetic biology or nephron development. We suggest either submitting the metabolic data in combination with the RNAseq data to a smaller journal, or significantly increasing the detail and amount of direct experimentation on NPs.

About the reviewer:
I am a senior post-doctoral fellow in the field of metabolic activity related to Acute Kidney Injury with a background in cellular and developmental biology. I do not have sufficient expertise to fully evaluate the bioinformatic data, however I have a thorough knowledge of the current literature in metabolic activity specifically related to the kidney. I also consulted extensively with my PI Dr. Sims-Lucas who is an expert in kidney development and metabolism of nephron progenitors and during kidney injury.

Reviewer 3

Evidence, reproducibility and clarity

Summary:

The paper by Known et al. shed light on an important and current theme on the field, which is what are the cues that govern NPC fate decisions during nephrogenesis. the authors attack this question with a dual approach based on RNA seq and Metabolomics. They found that MAPK/ERK signaling (through its role in cell metabolism) is essential for proper kidney development.The importance of MAPK/ERK for NPC niche regulation has already been demonstrated (by this same group). However, the metabolomics analysis brings new and interesting findings on the interplay between cell fate decisions and metabolism.

Major points:

1-the paper refers to a RNA seq experiment done in NPC isolated from E13.5 mouse embryos that will be published elsewhere. This makes difficult to analyze such results. We see heterogeneity E13.5 NPC in our lab.
It is not possible to see differences in NPC populations present at E13.5 with Bulk RNA seq. It would be better if the authors had done single cell RNA seq instead. Perhaps, the author could download one of the scRNA seq data available at GEO datasets and quickly compare the results with their own. The question to be addressed is: could the metabolic differences observed in this paper be particular to a sub population of NPCs within the NPC niche. (not a absolute requirement, but to be considered)
Do the author see any Wnt4 expression with their dataset? if yes how would it change the fidings? since Wnts may play roles in metabolism.

2-At the end of the second paragraph of the results section. The authors state that: " GO andKEGG dataset pathway demonstrated that mitochondrial inner membrane (25%) and mitochondrial (15%)

© 2022. Published by The Company of Biologists under the terms of the Creative Commons Attribution License (https://creativecommons.org/licenses/by/4.0/).

account 40% of the down-regulated transcript". Because the same pool of genes were used in KEGG and GO (cellular component), I wonder how much overlap there is here (do the same genes showing in both scores?), If genes were counted twice, (in both KEGG and GO), were only count in one base (either KEGG or GO) would it still be 40%?

3-The metabolomics analysis were performed in mK4 cells, after an in vitro culture for some time. The immortalized cell line, may not recapitulated the metabolic scenario of a naive NPC in its niche.
Also, the in vitro system affects the cellular metabolism (nutrients availbility, signals gradient etc). would it not be better to have used freshly isolated NPC for this experiment? That being said, metabolomics required a large number of cells, and it present itself as a problem, in terms of cell availability.

We have noticed that the interaction with UB and the adjacent stroma coordinate metabolic changes in the NPC pool. Perhaps, a better explanation of the limitations of the system would fit well in this paper, assuming the general reader may not be familiar with the complexity of the system or the techniques used.

4-It would be great if the author could show that pharmacological inhibition of MAPK/ERK affects cell metabolism with a functional assay such as seahorse respirometry Or mitochondrial respirometry with Oroboros respirometry assays. Or some type of functional assay

5-Pycr-1 has a role for the NADP metabolism, also PYCR-1 deficiency has been implicated in progeroid syndrome in humans (https://pubmed.ncbi.nlm.nih.gov/19648921). Would proline metabolism be the only aspect to be evaluated here? A better characterization regarding mitochondrial respiration and morphology in NPC lacking Pycr1 and 2 is needed. My guess is these cells are undergoing Oxidative stress. How the expression of oxidative stress response genes is affected in these KOs?

Minor points:

1-the reference list appears to have some mismatches with the text, please review it (e.g results section, second text line ref 20, is not the one the authors intended to cite)

2-metabolomics analisys of MAPK/ERK deficiency section. the authours say " comparisons of the untargeted MS2 spectrum patterns with a online MS database" please provide the reference of the database.

3-the gene lists in the the supplementary material appear to be out of bound regarding margins.

4-It would be nice if the authours could make the metabolomic and RNA seq data available with a web interactive tool, such as shiny web application or dashboard application. so, other scientists would be able to browse through the data.

Significance

The metabolomic analysis of NPC and the developing kidney in itself gives cues on what the cells are doing, whereas, RNA seq provide a idea on what the cell will do next. The combination of both is a powerful way to understand the complicate network of signals that drives cell fate decisions within the developing kidney.

I think this paper is suitable for publication after major changes. It is, nonetheless interesting see that people is starting to combine transcriptomics and metabolomics to have a better idea of fate decisions in progenitor cells.

© 2022. Published by The Company of Biologists under the terms of the Creative Commons Attribution License (https://creativecommons.org/licenses/by/4.0/).

Author response to reviewers' comments

Reviewer 1
Summary
In this manuscript Kwon et al use a multi-omics-based approach to identify the MAPK/ERK pathway as contributor to the maintenance and propagation of the developing kidney. This claim is first supported by transcriptional profiling of nephron progenitor cells with conditional inactivation of both Mek genes (Mek1/Mek2) which identifies >5000 differentially expressed genes with further analysis of these genes via RNA-Seq showing mitochondrial metabolism is most effected by MAPK/ERK inactivation. The physiological consequences are then examined via pyruvate titration experiments using E11.5 mouse embryonic kidneys which claimed to show reduced branching of the ureteric buds and double Pycr1 and Pycr2 knockouts revealing defects in NP as evidence by reduced PAX6 and SIX2 staining.

Major comments
-The transcriptomic profiling data and further figures identifying the MAPK/ERK pathway is contributing to the maintenance and propagation of nephron progenitor cells. However experiments showing physiological consequences need more detailed analysis. What could, for example, be done is using direct inhibition of MAPK/ERK via UO126 used in previous figures (Fig 2) to check for any morphological consequences on ureteric bud branching in embryonic kidney cultures and to compare against simple pyruvate titration experiments.

> We thank the reviewer for useful and constructive comments that have significantly improved our manuscript. We addressed the first comment in the revised manuscript by the following addition:
> *"We and others have previously published the effect of MEK inhibitor(s) on ureteric bud branching morphogenesis (Fisher et al., 2001; Hida et al., 2002; Ihermann-Hella et al., 2018). Moreover, our ureteric bud specific genetic experiments demonstrate very similar phenotype to that observed with chemical MEK inhibition (Ihermann-Hella et al., 2018). In essence, MAPK/ERK-deficient ureteric bud fails to generate complex branched epithelial structures. This is due to failure of ureteric bud tips, which contain the collecting duct progenitors, to expands and form an enlarged ampulla preceding the tip bifurcation required for new branch formation. The MAPK/ERK-deficient morphology resembles the ureteric bud morphology observed here in low pyruvate concentrations (Figure 6A) thus supporting our findings that MAPK/ERK regulates progenitors by contributing to their metabolic control."*

-Why is LC/MS analysis done on an embryonic kidney mesenchyme cell line and not on NPs isolated from E13.5 kidneys used for the RNA-seq analysis?

> The reviewer's comment is very relevant, but technically challenging. We have addressed this in the revised manuscript by the following addition and hope that the reviewer will appreciate the efforts and new information our approach has generated, especially as it is well supported by *in vivo* experimentation:
> *"Reliable metabolomic analysis requires a large number of cells, and this presents a challenge due to embryonic NP availability. Although these cells have been isolated and successfully propagated in vitro in nephrosphere cultures, our pilot experiments showed that their utilization for MAPK/EKR-regulated metabolic target study was not feasible due to the need for extensive use of growth factors, other proteins and competitive inhibitors (Brown et al., 2015; Li et al., 2016)".*

-Further comments on the morphological differences of proline depletion (Pycr1/2 dko) would also be valuable. There seems to be an inversion of expected phenotype particularly in images stained with SIX2. Maybe consider that lack of proline reduces NPC proliferation causing premature differentiation into a renal vesicle. Here, the paper could benefit from specific immunostainings against CDH6 or other epithelial markers.

> As suggested by the reviewer, we have performed additional characterization of *Pycr1/2* double knockout kidneys. This included immunofluorescent staining with cadherin6 (CDH6), a nephron differentiation marker that visualizes epithelializing nephrons from early pretubular aggregates to S-shaped bodies, where CDH6 localizes to proximal tubule progenitors (Cho et al., 1998). Furthermore, we have used cyclin D1 (renal vesicle) as additional differentiation commitment markers and to analyze whether polarization of early epithelializing structures and determination of nephron segment identities are affected.

© 2022. Published by The Company of Biologists under the terms of the Creative Commons Attribution License (https://creativecommons.org/licenses/by/4.0/).

Our new results (Fig. 7A-F) of revised manuscript) demonstrate that concomitantly to diminished amount of nephron progenitors, *Pycr1/2* kidneys show increase in nephron precursor structures.

The quantification of CDH6-positive nephron precursor structures demonstrates rise, which however is statistically not significant while the quantification of cyclin D1-positive renal vesicles, which represent more restricted precursor pool, shows statistically significant increase.

We also characterized the main cellular processes, proliferation and apoptosis, that contribute to nephron progenitor maintenance to establish whether these contribute to premature loss of nephron progenitors in *Pycr1/2* kidneys. This revealed that proliferation and apoptosis are not affected in *Pycr1/2* double knockout kidneys (Fig 7G and S1D-G).

Together the new results show that *in vivo* disruption of proline metabolism causes premature nephron progenitor exhaustion through their accelerated differentiation in pyrroline-5-carboxylate reductases 1 and 2 (*Pycr1/2*) double-knockout kidneys. This suggests that proline, likely together with interconnected pyruvate metabolism (see discussion in revised manuscript) contributes to the maintenance of undifferentiated status in nephron progenitor cells.

-Is there a potential connection between pyruvate and proline metabolism? If yes, please discuss.

We want to thank the reviewer for making this excellent point. Inspired by the comment, we explored this further and now provide following discussion in the revised manuscript:

"Our results indicate that MAPK/ERK-deficiency affects also both pyruvate and proline metabolism, which are interconnected with each other (Figure 5). Pyruvate, an end-product of glycolysis, is converted into acetyl-CoA to feed the mitochondrial tricarboxylic acid (TCA) cycle. Proline is mainly synthesized from glutamate during glutaminolysis, which has crucial compensatory functions under glucose deprivation conditions due to its ability to convert glutamine into glutamate (Wu et al., 2014). As glycolysis and glutaminolysis are the main energy metabolism pathways in mammals they both likely contribute to the energy deprivation in MAPK/ERK-deficient cells."

"It has been shown that under pyruvate deprivation, glutaminolysis increases the concentration of mitochondrial glutamate, which can be converted into pyrroline 5-carboxylate (P5C), an intermediate metabolic product that contributes to both biosynthesis and catabolism of proline (Yang et al., 2014). Since proline is mainly synthesized from glutamate or ornithine (Jones, 1985), diminished mitochondrial glutamate stemming from the poorly functioning glutaminolysis may directly affect P5C production. This together with down-regulated pyrroline-5-carboxylate reductases (Pycr1/l) likely contributes to the reduced proline synthesis in MAPK/ERK-deficient cells (Figure 5)."

<u>Minor comments</u>

•Images from figure 6a and 6b showing physiological consequences of pyruvate concentration on embryonic kidneys do not have appropriate scale bars

•Would be good to have statistical analysis for figure 6c to show if the differences between WT and dko lines are statistically significant.

We thank the reviewer pointing out these shortages in the original manuscript. We have now added the missing scale bars to the figure 6A-B images of revised manuscript. We also included new data figure showing the statistical test results (Figure 6C).

•Would be good to have the information about sample/number of replicates on the graph.

The details of sample numbers used for analyses were originally included in the methods section of our manuscript. We have now included this information also to the figure legends to make it easier to evaluate the results.

Reviewer 2

<u>Summary:</u>

In the manuscript by Kwon et al a deeper understanding of metabolic changes associated with nephron progenitor differentiation is achieved through multi-disciplinary analysis. Mass spectrometry of a cell line representing normal nephron progenitor behavior in comparison with RNA-seq data brought the researchers to focus specifically on the MAPK/ERK pathway as one of interest in determining the pluripotent or differentiated status of NPs. They tested the effects of

© 2022. Published by The Company of Biologists under the terms of the Creative Commons Attribution License (https://creativecommons.org/licenses/by/4.0/).

MAPK/ERK deficiency on the downstream process of proline metabolism and specifically the genes PYCR1/2 in vivo, both with organ culture and a knockout mouse line. While these in vivo studies did not show a gross phenotype associated with defective embryonic development, the authors conclude that a tightly controlled level of pyruvate and PYCR1/2 activity is necessary to prevent premature differentiation of nephron progenitors.
7. Additional markers of NP differentiation and increased number of samples quantified in Figure 6 would strengthen the results

Criticisms:
1.Overall, this reviewer agrees that the conclusions drawn, while speculative, are factually correct based on the experimentation. However, the experiments performed (especially in vivo) fall short of determining any major evidence on the metabolism of nephron progenitors, and thus this reviewer does not believe that the preliminary findings are significant enough to progress the field.

We thank the reviewer for his/her opinion on our work, which we obviously don't fully agree. Our previous study has demonstrated the *in vivo* importance of MAPK/ERK pathway for nephron progenitor maintenance and differentiation (Ihermann-Hella et al., 2018). Here we further studied the mechanisms through which MAPK/ERK activity maintains nephron progenitors in developing kidney. In the initial manuscript we had performed and analyzed transcriptomic and metabolic characterization of MAPK/ERK-deficient cells *in vivo* and *in vitro*. Functional validation of identified metabolic changes included *in vitro* studies of pyruvate effects on kidney growth and ureteric bud morphology and generation of new double knockout mouse model of *Pycr1/2*-deficiency, which *in vivo* renal phenotype showed failure to maintain postnatal nephron progenitors.

In the revised manuscript we provide more in depth analysis of our RNA-seq results and metabolic profiling. The new data is presented in figures 4, 6C, 7 and S1, and tables S2 and S3 of the revised manuscript. Based on this we can conclude that MAPK/ERK-activity regulates

1) energy preference of nephron progenitors, which is supported by previous work demonstrating this with one of the MAPK/ERK targets p53 (Li et al., 2015) and our work now published at BMC Biology (Kurtzeborn et al., 2022).
2) ribosome biogenesis, which is directly interlinked with proline metabolism but also more generally reflects the identified alterations in amino acid metabolism (Krafczyk et al., 2021; Melnikov et al., 2016).
3) proline metabolism, which is important factor influencing nephron progenitor fate decisions

Amino acid metabolism, including glutamine, polyamines, and tryptophan, is an established critical regulator of stemness in pluripotent stem cells but its contribution to tissue-specific progenitor cell regulation is only emerging (Allmeroth et al., 2021; Kim et al., 2020; Someya et al., 2021; Zhao et al., 2012). Very recently methionine metabolism was suggested to contribute to NPs proliferation (Makayes et al., 2021) further validating our findings.

We hope that the reviewer will appreciate that by identifying the metabolites that are affected by nephron progenitor regulator MAPK/ERK pathway we are not only providing important missing directions for the future studies, but have here validated some of their *in vivo* and *in vitro* effects.

2.The metabolomics data and RNA seq data (while not specifically provided in this manuscript, but appears to be in preparation for a separate manuscript) are no doubt solid and interesting, they fail to be properly translated into cellular behavior or mechanism.

We have now amended our manuscript with additional experimental data and provide the accession number of our published RNAseq raw data at GEO (*https://www.ncbi.nlm.nih.gov/geo/query/acc.cgi?acc=GSE174229*). Thanks to the reviewer comments, the revised manuscript now translates our omics findings to more in-depth mechanistic view of MAPK/ERK-dependent metabolic processes in nephron progenitor regulation.

3. The lack of a phenotype in the knockout mouse leaves the reader unconvinced of how this pathway is a "gatekeeper". We suggest that the paper be supported with significantly more in vivo experiments including various time points and more defined read-outs of metabolic activity.

© 2022. Published by The Company of Biologists under the terms of the Creative Commons Attribution License (https://creativecommons.org/licenses/by/4.0/).

It is difficult to catch which mouse model the reviewer is referring to with this comment. We have previously genetically demonstrated the importance of MAPK/ERK activity in nephron progenitor regulation (Ihermann-Hella et al., 2018). We showed that MAPK/ERK activity plays dual functions during nephrogenesis; it is required for nephron progenitor self-renewal and their timely differentiation. Here we studied the transcriptional and metabolic changes MAPK/ERK-deficiency induces in nephron progenitors. We additionally show by *in vivo* genetic model (*Pycr1/2* double knockout kidneys) that disruption of proline metabolism results in reduced nephron progenitor amount in newborn kidneys. In the title of our manuscript gatekeeper refers to MAPK/ERK activity and there is experimental evidence presented in our manuscript which supports this conclusion

4.The LC/MS was performed on a cell line, rather than in vivo, which clouds the results especially since the specific cell line is never characterized in terms of timing of development, genetic changes such a immortalization etc.

The reviewer's comment is very relevant, but technically challenging. We have addressed this in the revised manuscript by the following addition and hope that the reviewer will appreciate the efforts and new information our approach has generated, especially as it is well supported by *in vivo* experimentation:

"Reliable metabolomic analysis requires a large number of cells, and this presents a challenge due to embryonic NP availability. Although these cells have been isolated and successfully propagated in vitro in nephrosphere cultures, our pilot experiments showed that their utilization for MAPK/EKR-regulated metabolic target study was not feasible due to the need for extensive use of growth factors, other proteins and competitive inhibitors (Brown et al., 2015; Li et al., 2016)".

5.Figure 4 is difficult to understand as it seems unfocused, underlining a lack of specific direction in the study of metabolics.

The aim of the figure 4 is to demonstrate the different metabolic pathways mutually affected by MAPK/ERK activity both at mRNA and metabolite level. We interpret this so that it gives higher plausibility and thus stronger support for the identified change. We do agree that due to the changes in many parallel and distinct metabolic pathways this may seem rather complicated. To better align with our overall message, we have reorganized and amended the revised figure 4 as follows: to group the identified changes: Amino acid metabolism, Aspartate metabolism, ATP related, Citrate metabolism, Glutathione synthesis, Glutaminolysis, Glycine metabolism, Glycolysis and Proline metabolism. We hope that these amends will make the figure easier to understand.

6.We strongly suggest that different time points be tested in a single experiment to prove a defect rather than a delay

Unfortunately, this comment is impossible for us to understand without further specifications. Assuming that the reviewer is referring to analysis of different time points in *Pycr1/2* knockout kidneys we find this suggestion technically possible but not really addressing anything that would "prove a defect rather than a delay".

Our data suggests that nephron progenitors are prematurely lost due to accelerated differentiation in postnatal kidneys of *Pycr1/2* knockout pups. This can by no means derive from a delay, but rather is caused by insufficient proline production that causes either nephron progenitor death, defective propagation, premature differentiation or any combination of these cellular events.

As show by our new results (Fig. 7, S1D-G, S2), proliferation and apoptosis are not affected in *Pycr1/2* double knockout kidneys but. Instead, our results show that *in vivo* disruption of proline metabolism causes premature nephron progenitor exhaustion through their accelerated differentiation. This suggests that proline, likely together with interconnected pyruvate metabolism (see discussion in revised manuscript and below) contributes to the maintenance of undifferentiated status in nephron progenitor cells.

7.Additional markers of NP differentiation and increased number of samples quantified in Figure 6 would strengthen the results

As suggested by the reviewer, we have performed additional characterization of *Pycr1/2* double knockout kidneys. This included immunofluorescent staining with cadherin6 (CDH6), a nephron differentiation marker that visualizes epithelializing nephrons from early pretubular

© 2022. Published by The Company of Biologists under the terms of the Creative Commons Attribution License (https://creativecommons.org/licenses/by/4.0/).

aggregates to S-shaped bodies, where CDH6 localizes to proximal tubule progenitors (Cho et al., 1998). Furthermore, we have used cyclin D1 (renal vesicle) as additional differentiation commitment markers and to analyze whether polarization of early epithelializing structures and determination of nephron segment identities are affected.
Our new results (Fig. 7A-F) of revised manuscript) demonstrate that concomitantly to diminished amount of nephron progenitors, *Pycr1/2* kidneys show increase in nephron precursor structures. The quantification of CDH6-positive nephron precursor structures demonstrates rise, which however is statistically not significant while the quantification of cyclin D1-positive renal vesicles, which represent more restricted precursor pool, shows statistically significant increase.

We also characterized the main cellular processes, proliferation and apoptosis, that contribute to nephron progenitor maintenance to establish whether these contribute to premature loss of nephron progenitors in *Pycr1/2* kidneys. This revealed that proliferation and apoptosis are not affected in *Pycr1/2* double knockout kidneys (Fig 7G and S1D-G).

Together the new results show that *in vivo* disruption of proline metabolism causes premature nephron progenitor exhaustion through their accelerated differentiation in pyrroline-5-carboxylate reductases 1 and 2 (*Pycr1/2*) double-knockout kidneys. This suggests that proline, likely together with interconnected pyruvate metabolism (see discussion in revised manuscript) contributes to the maintenance of undifferentiated status in nephron progenitor cells.

8.Neither scale bars or statistical testing were shown in Figure 6 and the images in Figure 6B are shown at inconsistent magnifications

We added the missing scale bars to the figure 6A-B images of revised manuscript. These figure images are of the same magnification. We also included the statistical test results to the figure 6C and made the Y axes to match each other.

9.There are several areas of the text that need adequate proofreading including typos and syntax. It is very clear that several people contributed to the manuscript leaving the reader with a very piecemeal combination of authors voices.

We have initially tried our best to use English language appropriately and the second author of the manuscript is actually a native speaker, who also proof-read the text before submission. The revised manuscript is again proof-red not only for spelling mistakes and syntax but also for coherence. To improve coherence, we also deleted some parts of the discussion which is not relevant for our main messages. All these changes are shown in blue/track changes to make it easier for the editors and reviewers to identify modified sections.

Reviewer 3

Summary:

The paper by Known et al. shed light on an important and current theme on the field, which is what are the cues that govern NPC fate decisions during nephrogenesis. the authors attack this question with a dual approach based on RNA seq and Metabolomics. They found that MAPK/ERK signaling (through its role in cell metabolism) is essential for proper kidney development. The importance of MAPK/ERK for NPC niche regulation has already been demonstrated (by this same group). However, the metabolomics analysis brings new and interesting findings on the interplay between cell fate decisions and metabolism.

Major points:

1-the paper refers to a RNA seq experiment done in NPC isolated from E13.5 mouse embryos that will be published elsewhere. This makes difficult to analyze such results. We see heterogeneity E13.5 NPC in our lab.

We thank the reviewer for useful and constructive comments that have significantly improved our manuscript.
We have now published the transcriptomics data (Kurtzeborn et al., 2022) and (*https://www.ncbi.nlm.nih.gov/geo/query/acc.cgi?acc=GSE174229*) and amended the manuscript accordingly .

© 2022. Published by The Company of Biologists under the terms of the Creative Commons Attribution License (https://creativecommons.org/licenses/by/4.0/).

1-It is not possible to see differences in NPC populations present at E13.5 with Bulk RNA seq. It would be better if the authors had done single cell RNA seq instead. Perhaps, the author could download one of the scRNA seq data available at GEO datasets and quickly compare the results with their own. The question to be addressed is: could the metabolic differences observed in this paper be particular to a sub population of NPCs within the NPC niche. (not a absolute requirement, but to be considered)

> We are fully aware of the methodological limitations of bulk RNA-seq, and appreciate the fact that NPCs are heterogenous population consisting of multiple sub-types of progenitor cells, each of which could contribute to the observed differential gene expressions. Moreover, we have previously demonstrated different MAPK/ERK activation modes among individual MM cells in the developing kidney (Ihermann-Hella et al., 2018). Studies in other models have demonstrated that depending on the duration and the type of stimuli, these result in either sustained or dynamic MAPK/ERK activation (Catalanotti et al., 2009; Kamioka et al., 2012; Kumagai et al., 2015). Studies in other systems have additionally demonstrated that the transcriptional responses and cell fate decisions affected by the type and strength of MAPK/ERK activation are tissue specific (De et al., 2020; Johnson and Toettcher, 2019; Wilson et al., 2017). Pulsatile ERK activation causes different transcriptional responses and cell fate decisions in early *Drosophila* embryo than what it does in normal skin epithelial and epithelial cancer cells (De et al., 2020; Dumesic et al., 2009; Johnson and Toettcher, 2019). Therefore, our future aim is to combine powerful new types of scRNA-seq with our previously used mouse line visualizing ERK by FRET-biosensor to profile differences in transcriptional responses among developing kidney cells having heterogeneous levels of MAP/ERK activation. Having here done the bulk RNA-seq will allow comparison of those results to the bulk RNA-seq results further defining the mechanisms through which MAPK/ERK operates in developing kidney.
>
> Here, as suggested by the reviewer, we compared the key transcriptional changes identified upon MAPK/ERK-deficiency to single cell RNA-seq dataset available at GEO with the accession number GSE59127 (Brunskill et al., 2014). The nephron progenitors were divided to sub-populations based on their expression of *Six2*, *Cited1* and *Pax2*. The results of this analysis are presented in Results section chapter "Integration of metabolomics and RNA-Seq data suggests dysregulation in nephron progenitor fate decisions" and table S2 of revised manuscript. In essence, the data demonstrate increased representation of MAPK/ERK targets in the most stem-like (Six2+/Cited1+ double positive) nephron progenitor population.

1-Do the author see any Wnt4 expression with their dataset? if yes how would it change the fidings? since Wnts may play roles in metabolism.

> Some Wnt pathway transcripts were identified among MAPK/ERK-dependent differentially expressed genes. These include *Wnt4* (-1,74), *Fzd2* (-1,06), suggesting decreased differentiation signaling (Stark et al., 1994; Tanigawa et al., 2011) while modest upregulation of other receptors *Fzd1* and *-3* (1,18 and 1,04) together with several *Tcf* genes being both up- and downregulated were identified. Due to complex up- and downregulation findings it is impossible provide clear outcome of Wnt signaling changes and their possible connection to metabolism.

2-At the end of the second paragraph of the results section. The authors state that: " GO and KEGG dataset pathway demonstrated that mitochondrial inner membrane (25%) and mitochondrial (15%) account 40% of the down-regulated transcript". Because the same pool of genes were used in KEGG and GO (cellular component), I wonder how much overlap there is here (do the same genes showing in both scores?), If genes were counted twice, (in both KEGG and GO), were only count in one base (either KEGG or GO) would it still be 40%?

> We apologize for the confusing phrasing, which is now modified in the revised manuscript. In essence, the ontology source for mitochondrial inner membrane and mitochondrion is GO (cellular component), not KEGG. Among total 59 of GO Terms from down-regulated DEGs, only 13 GO Terms are identified through KEGG and the remaining 46 GO Terms are identified through GO cellular component. As shown in Table S1, none of GO Terms are overlapping.

3-The metabolomics analysis were performed in mK4 cells, after an in vitro culture for some time. The immortalized cell line, may not recapitulated the metabolic scenario of a naive NPC in its niche. Also, the in vitro system affects the cellular metabolism (nutrients availbility, signals gradient etc). would it not be better to have used freshly isolated NPC for this experiment? That

© 2022. Published by The Company of Biologists under the terms of the Creative Commons Attribution License (https://creativecommons.org/licenses/by/4.0/).

being said, metabolomics required a large number of cells, and it present itself as a problem, in terms of cell availability.

The reviewer's comment is very relevant, but technically challenging. We have addressed this in the revised manuscript by the following addition and hope that the reviewer will appreciate the efforts and new information our approach has generated, especially as it is well supported by *in vivo* experimentation:

"Reliable metabolomic analysis requires a large number of cells, and this presents a challenge due to embryonic NP availability. Although these cells have been isolated and successfully propagated in vitro in nephrosphere cultures, our pilot experiments showed that their utilization for MAPK/EKR-regulated metabolic target study was not feasible due to the need for extensive use of growth factors, other proteins and competitive inhibitors (Brown et al., 2015; Li et al., 2016)".

3-We have noticed that the interaction with UB and the adjacent stroma coordinate metabolic changes in the NPC pool. Perhaps, a better explanation of the limitations of the system would fit well in this paper, assuming the general reader may not be familiar with the complexity of the system or the techniques used.

This important comment is addressed in introduction and discussion sections of the revised manuscript as follows:

Introduction: "*Together, the UB, stromal cells in the most cortical part of developing kidney, and MM form the NP niche, where balanced maintenance and propagation of NPs occur (Oxburgh, 2018). The complex interplay of signals originating from all niche compartments maintains their cell identities without much of time-dependent changes in niche composition but with clear differences in cell behaviors and molecular players."*

Discussion: *"In this study we focused on understanding the MAPK/ERK-dependent transcriptional and metabolic control of NP cell maintenance, propagation, and differentiation. For this, NP cells with intact or depleted MAPK/ERK activity were utilized. This strategy is a powerful way to study NP niche intrinsic mechanisms in nephrogenesis guidance. However, it bypasses the biological complexity of whole kidney morphogenesis regulation as the functions of ureteric bud epithelium and stroma, which contribute to NP niche composition are ignored. We and others have shown that several ureteric bud derived signals significantly contribute to NP cell biology (Karner et al., 2009; Li et al., 2021; Lindstrom et al., 2015)and the function of stromal signals is emerging (Naiman et al., 2017; Zhang et al., 2019). Thus, further studies are needed to build up holistic view on how nephron progenitor metabolism integrates to the guidance of optimally functioning kidney."*

4-It would be great if the author could show that pharmacological inhibition of MAPK/ERK affects cell metabolism with a functional assay such as seahorse respirometry Or mitochondrial respirometry with Oroboros respirometry assays. Or some type of functional assay

We have both measured mitochondrial counts per cell and adenosine triphosphate (ATP) availability as well as performed Seahorse metabolic profiling in the absence of MAPK/ERK activation. These results are now published at BMC Biology (Kurtzeborn et al., 2022). In essence, these experiments demonstrate

1. Mitochondria amount per cell, as determined by mtDNA copy number against nuclear DNA copy number, is statistically significantly reduced
2. analytic quantification of ATP amount by liquid chromatography-mass spectrometry (LC/MS) shows significant decrease
3. Basal respiration, maximal respiration and spare capacity are all diminished as measured by Seahorse

NOTE: *Figure provided for reviewer has been removed. It showed part of Figure 6 from Kurtzeborn et al. (2022) Comparative whole-genome transcriptome analysis in renal cell populations reveals high tissue specificity of MAPK/ERK targets in embryonic kidney. BMC Biol 20, 112 (DOI: 10.1186/s12915-022-01309-z).*

Figure 6 of Kurtzeborn paper (Kurtzeborn et al., 2022) (**B**) Mitochondrial DNA copy number analysis was performed on control (DMSO, n=9) and MEK1/2-inhibited (U0126, n=9) E12.5 kidneys by real-time PCR. Mitochondrial DNA was measured by the analysis of its 12S expression against nuclear DNA quantification by *Rbm* expression. (**C**) Quantification of ATP

© 2022. Published by The Company of Biologists under the terms of the Creative Commons Attribution License (https://creativecommons.org/licenses/by/4.0/).

from mK4 cell line derived from embryonic kidney mesenchyme by LC/MS (n=4 for each DMSO and U0126). ATP concentrations are normalized against total protein concentration. ** = p < 5 * 10-3, *** = p < 5 * 10-6. (**D**) Oxygen consumption rate (OCR) of mK4 cell line was measured by a Seahorse XF analyzer. For the OCR measuring, ATP synthase inhibitor (Oligomycin), protonophore uncoupler (FCCP), and ETC inhibitors (Rotenone and Antimycin A) were added at the indicated points. (**E**) Basal respiration, ATP production, proton leak, maximal respiration, and spare capacity measures are shown in different samples. Error bars represent standard deviation (S.D.) NT; non-treated mK4 cells, DMSO; DMSO-treated mK4 cells as a control, U0126; MEK inhibitor U0126-treated mK4 cells.

5-Pycr-1 has a role for the NADP metabolism, also PYCR-1 deficiency has been implicated in progeroid syndrome in humans (https://pubmed.ncbi.nlm.nih.gov/19648921). Would proline metabolism be the only aspect to be evaluated here? A better characterization regarding mitochondrial respiration and morphology in NPC lacking Pycr1 and 2 is needed. My guess is these cells are undergoing Oxidative stress. How the expression of oxidative stress response genes is affected in these KOs?

The effect of Pycr1 and -2 on mitochondrial integrity and functions as well as oxidative stress seem to be very cell-type specific (Escande-Beillard et al., 2020; Reversade et al., 2009; Stum et al., 2021). As suggested, we have performed additional experiments to examine how defective proline metabolism affects cellular behaviors of nephron progenitors. This includes analysis of nephron progenitor propagation, apoptosis (studied by cleaved-Caspase 3) and differentiation (Figures 7 and S1 of revised manuscript) as well as mitochondrial morphology in *Pyrc1/2* double knockout kidneys (Figure S2 of revised manuscript).

These experiments revealed that unlike in mouse central nervous system and in *Xenopus* and zebrafish skin (Escande-Beillard et al., 2020; Reversade et al., 2009), NAD(P)H-dependent conversion of pyrroline-5-carboxylate to proline in nephron progenitor cells does not induce cell death (Figure S1). Similarly to what is reported *in vivo* (Stum et al., 2021), loss of *Pycr1/2* did not grossly alter the mitochondrial network nephron progenitors (Figure S2), which is opposite what is reported *in vitro* (Kuo et al., 2016). As oxidative stress has not been recapitulated in mammalian models of PYCR-deficiency we did not have a chance to analyze this in *Pycr1/2* double knockout kidneys.

Inspired by the reviewer’s comment we also examined oxidative stress related transcriptional signatures in our RNA-seq dataset. These results are presented in the table S3 of revised manuscript. In essence, these results suggest perturbed antioxidant defense, which may contribute to the diminished cell cycle progression in MAPK/ERK-deficient NPs without increasing cell death.

Minor comments

1-the reference list appears to have some mismatches with the text, please review it (e.g results section, second text line ref 20, is not the one the authors intended to cite)

We appreciate for pointing this out. The reference list is now updated in the revised manuscript.

2-metabolomics analisys of MAPK/ERK deficiency section. the authours say " comparisons of the untargeted MS2 spectrum patterns with a online MS database" please provide the reference of the database.

As requested, the revised manuscript now included reference to the MS database.

3-the gene lists in the the supplementary material appear to be out of bound regarding margins.

The supplementary tables are now included as original excel file to avoid any inconvenience in their reading.

4-It would be nice if the authours could make the metabolomic and RNA seq data available with a web interactive tool, such as shiny web application or dashboard application. so, other scientists would be able to browse through the data.

The RNAseq raw data is publicly available at GEO https://www.ncbi.nlm.nih.gov/geo/ under the accession number GSE174229 and our metabolites are included here. Although

© 2022. Published by The Company of Biologists under the terms of the Creative Commons Attribution License (https://creativecommons.org/licenses/by/4.0/).

these do not make data browsing as easy as interactive web-based tool would, we feel that building such would lead to an entire new article.

Allmeroth, K., Kim, C. S., Annibal, A., Pouikli, A., Koester, J., Derisbourg, M. J., Andres Chacon-Martinez, C., Latza, C., Antebi, A., Tessarz, P., et al. (2021). N1-acetylspermidine is a determinant of hair follicle stem cell fate. *J Cell Sci* **134**.

Brown, A. C., Muthukrishnan, S. D. and Oxburgh, L. (2015). A synthetic niche for nephron progenitor cells. *Dev Cell* **34**, 229-241.

Brunskill, E. W., Park, J. S., Chung, E., Chen, F., Magella, B. and Potter, S. S. (2014). Single cell dissection of early kidney development: multilineage priming. *Development* **141**, 3093-3101.

Catalanotti, F., Reyes, G., Jesenberger, V., Galabova-Kovacs, G., de Matos Simoes, R., Carugo, O. and Baccarini, M. (2009). A Mek1-Mek2 heterodimer determines the strength and duration of the Erk signal. *Nat Struct Mol Biol* **16**, 294-303.

Cho, E., Patterson, L., Brookhiser, W., Mah, S., Kintner, C. and Dressler, G. (1998). Differential expression and function of cadherin-6 during renal epithelium development. *Development* **125**, 803-812.

De, S., Campbell, C., Venkitaraman, A. R. and Esposito, A. (2020). Pulsatile MAPK Signaling Modulates p53 Activity to Control Cell Fate Decisions at the G2 Checkpoint for DNA Damage. *Cell reports* **30**, 2083-2093 e2085.

Dumesic, P. A., Scholl, F. A., Barragan, D. I. and Khavari, P. A. (2009). Erk1/2 MAP kinases are required for epidermal G2/M progression. *J Cell Biol* **185**, 409-422.

Escande-Beillard, N., Loh, A., Saleem, S. N., Kanata, K., Hashimoto, Y., Altunoglu, U., Metoska, A., Grandjean, J., Ng, F. M., Pomp, O., et al. (2020). Loss of PYCR2 Causes Neurodegeneration by Increasing Cerebral Glycine Levels via SHMT2. *Neuron* **107**, 82-94 e86.

Fisher, C. E., Michael, L., Barnett, M. W. and Davies, J. A. (2001). Erk MAP kinase regulates branching morphogenesis in the developing mouse kidney. *Development* **128**, 4329-4338.

Hida, M., Omori, S. and Awazu, M. (2002). ERK and p38 MAP kinase are required for rat renal development. *Kidney Int* **61**, 1252-1262.

Ihermann-Hella, A., Hirashima, T., Kupari, J., Kurtzeborn, K., Li, H., Kwon, H. N., Cebrian, C., Soofi, A., Dapkunas, A., Miinalainen, I., et al. (2018). Dynamic MAPK/ERK Activity Sustains Nephron Progenitors through Niche Regulation and Primes Precursors for Differentiation. *Stem Cell Reports*.

Johnson, H. E. and Toettcher, J. E. (2019). Signaling Dynamics Control Cell Fate in the Early Drosophila Embryo. *Dev Cell* **48**, 361-370 e363.

Jones, M. E. (1985). Conversion of glutamate to ornithine and proline: pyrroline-5-carboxylate, a possible modulator of arginine requirements. *J Nutr* **115**, 509-515.

Kamioka, Y., Sumiyama, K., Mizuno, R., Sakai, Y., Hirata, E., Kiyokawa, E. and Matsuda, M. (2012). Live imaging of protein kinase activities in transgenic mice expressing FRET biosensors. *Cell Struct Funct* **37**, 65-73.

Karner, C. M., Chirumamilla, R., Aoki, S., Igarashi, P., Wallingford, J. B. and Carroll, T. J. (2009). Wnt9b signaling regulates planar cell polarity and kidney tubule morphogenesis. *Nat Genet*.

Kim, C. S., Ding, X., Allmeroth, K., Biggs, L. C., Kolenc, O. I., L'Hoest, N., Chacon-Martinez, C. A., Edlich-Muth, C., Giavalisco, P., Quinn, K. P., et al. (2020). Glutamine Metabolism Controls Stem Cell Fate Reversibility and Long-Term Maintenance in the Hair Follicle. *Cell Metab* **32**, 629-642 e628.

Krafczyk, R., Qi, F., Sieber, A., Mehler, J., Jung, K., Frishman, D. and Lassak, J. (2021). Proline codon pair selection determines ribosome pausing strength and translation efficiency in bacteria. *Commun Biol* **4**, 589.

Kumagai, Y., Naoki, H., Nakasyo, E., Kamioka, Y., Kiyokawa, E. and Matsuda, M. (2015). Heterogeneity in ERK activity as visualized by in vivo FRET imaging of mammary tumor cells developed in MMTV-Neu mice. *Oncogene* **34**, 1051-1057.

Kuo, M. L., Lee, M. B., Tang, M., den Besten, W., Hu, S., Sweredoski, M. J., Hess, S., Chou, C. M., Changou, C. A., Su, M., et al. (2016). PYCR1 and PYCR2 Interact and Collaborate with RRM2B to Protect Cells from Overt Oxidative Stress. *Sci Rep* **6**, 18846.

Kurtzeborn, K., Kwon, H. N., Iaroshenko, V., Faisal, I., Ambroz, M., Jin, X., Qureshi, T., Kupari, J., Ihermann-Hella, A., Vaananen, J., et al. (2022). Comparative whole-genome

© 2022. Published by The Company of Biologists under the terms of the Creative Commons Attribution License (https://creativecommons.org/licenses/by/4.0/).

transcriptome analysis in renal cell populations reveals high tissue specificity of MAPK/ERK targets in embryonic kidney. *BMC Biol* **20**, 112.

Li, H., Kurtzeborn, K., Kupari, J., Gui, Y., Siefker, E., Lu, B., Matlik, K., Olfat, S., Montano-Rodriguez, A. R., Huh, S. H., et al. (2021). Postnatal prolongation of mammalian nephrogenesis by excess fetal GDNF. *Development* **148**.

Li, Y., Liu, J., Li, W., Brown, A., Baddoo, M., Li, M., Carroll, T., Oxburgh, L., Feng, Y. and Saifudeen, Z. (2015). p53 Enables metabolic fitness and self-renewal of nephron progenitor cells. *Development* **142**, 1228-1241.

Li, Z., Araoka, T., Wu, J., Liao, H. K., Li, M., Lazo, M., Zhou, B., Sui, Y., Wu, M. Z., Tamura, I., et al. (2016). 3D Culture Supports Long-Term Expansion of Mouse and Human Nephrogenic Progenitors. *Cell Stem Cell*.

Lindstrom, N. O., Carragher, N. O. and Hohenstein, P. (2015). The PI3K pathway balances self-renewal and differentiation of nephron progenitor cells through beta-catenin signaling. *Stem Cell Reports* **4**, 551-560.

Makayes, Y., Resnick, E., Hinden, L., Aizenshtein, E., Shlomi, T., Kopan, R., Nechama, M. and Volovelsky, O. (2021). Increasing mTORC1 Pathway Activity or Methionine Supplementation during Pregnancy Reverses the Negative Effect of Maternal Malnutrition on the Developing Kidney. *J Am Soc Nephrol* **32**, 1898-1912.

Melnikov, S., Mailliot, J., Rigger, L., Neuner, S., Shin, B. S., Yusupova, G., Dever, T. E., Micura, R. and Yusupov, M. (2016). Molecular insights into protein synthesis with proline residues. *EMBO Rep* **17**, 1776-1784.

Naiman, N., Fujioka, K., Fujino, M., Valerius, M. T., Potter, S. S., McMahon, A. P. and Kobayashi, A. (2017). Repression of Interstitial Identity in Nephron Progenitor Cells by Pax2 Establishes the Nephron-Interstitium Boundary during Kidney Development. *Dev Cell* **41**, 349-365 e343.

Oxburgh, L. (2018). Kidney Nephron Determination. *Annu Rev Cell Dev Biol* **34**, 427-450.

Reversade, B., Escande-Beillard, N., Dimopoulou, A., Fischer, B., Chng, S. C., Li, Y., Shboul, M., Tham, P. Y., Kayserili, H., Al-Gazali, L., et al. (2009). Mutations in PYCR1 cause cutis laxa with progeroid features. *Nat Genet* **41**, 1016-1021.

Someya, S., Tohyama, S., Kameda, K., Tanosaki, S., Morita, Y., Sasaki, K., Kang, M. I., Kishino, Y., Okada, M., Tani, H., et al. (2021). Tryptophan Metabolism Regulates Proliferative Capacity of Human Pluripotent Stem Cells. *iScience* **24**, 102090.

Stark, K., Vainio, S., Vassileva, G. and McMahon, A. P. (1994). Epithelial transformation of metanephric mesenchyme in the developing kidney regulated by Wnt-4. *Nature* **372**, 679-683.

Stum, M. G., Tadenev, A. L. D., Seburn, K. L., Miers, K. E., Poon, P. P., McMaster, C. R., Robinson, C., Kane, C., Silva, K. A., Cliften, P. F., et al. (2021). Genetic analysis of Pycr1 and Pycr2 in mice. *Genetics* **218**.

Tanigawa, S., Wang, H., Yang, Y., Sharma, N., Tarasova, N., Ajima, R., Yamaguchi, T. P., Rodriguez, L. G. and Perantoni, A. O. (2011). Wnt4 induces nephronic tubules in metanephric mesenchyme by a non-canonical mechanism. *Dev Biol* **352**, 58-69.

Wilson, M. Z., Ravindran, P. T., Lim, W. A. and Toettcher, J. E. (2017). Tracing Information Flow from Erk to Target Gene Induction Reveals Mechanisms of Dynamic and Combinatorial Control. *Mol Cell* **67**, 757-769 e755.

Wu, H., Li, Z., Yang, P., Zhang, L., Fan, Y. and Li, Z. (2014). PKM2 depletion induces the compensation of glutaminolysis through beta-catenin/c-Myc pathway in tumor cells. *Cell Signal* **26**, 2397-2405.

Yang, C., Ko, B., Hensley, C. T., Jiang, L., Wasti, A. T., Kim, J., Sudderth, J., Calvaruso, M. A., Lumata, L., Mitsche, M., et al. (2014). Glutamine oxidation maintains the TCA cycle and cell survival during impaired mitochondrial pyruvate transport. *Mol Cell* **56**, 414-424.

Zhang, H., Bagherie-Lachidan, M., Badouel, C., Enderle, L., Peidis, P., Bremner, R., Kuure, S., Jain, S. and McNeill, H. (2019). FAT4 Fine-Tunes Kidney Development by Regulating RET Signaling. *Dev Cell* **48**, 780-792 e784.

Zhao, T., Goh, K. J., Ng, H. H. and Vardy, L. A. (2012). A role for polyamine regulators in ESC self-renewal. *Cell Cycle* **11**, 4517-4523.

© 2022. Published by The Company of Biologists under the terms of the Creative Commons Attribution License (https://creativecommons.org/licenses/by/4.0/).

Original submission

First decision letter

MS ID#: DEVELOP/2022/200986

MS TITLE: Omics profiling identifies MAPK/ERK pathway as a gatekeeper of nephron progenitor metabolism

AUTHORS: Hyuk Nam Kwon, Kristen Kurtzeborn, Vladislav Iaroshenko, Xing Jin, Abigail Loh, Nathalie Escande-Beillard, Bruno REVERSADE, Sunghyouk Park, and Satu Kuure

I have now heard back from two out of the three reviewers who originally reviewed your manuscript, and have reached a decision. The referees' comments are appended below, or you can access them online: please go to BenchPress and click on the 'Manuscripts with Decisions' queue in the Author Area.

The overall evaluation is positive and we would like to publish a revised manuscript in Development, but I would ask you to address the final comment from one of the reviewers regarding the extrapolations made from profiling cell lines. In the revised version, you should either provide metabolomics data in bona fide NP cells or tone down statements/conclusions accordingly, including revising the title as suggested and considering the data in the publication mentioned by the reviewer.

Reviewer 1

Advance summary and potential significance to field

In the manuscript by Kwon et al a deeper understanding of metabolic changes associated with nephron progenitor differentiation is achieved through multi-disciplinary analysis. Mass spectrometry of a cell line representing normal nephron progenitor behavior in comparison with RNA-seq data brought the researchers to focus specifically on the MAPK/ERK pathway as one of interest in determining the pluripotent or differentiated status of NPs. They tested the effects of MAPK/ERK deficiency on the downstream process of proline metabolism and specifically the genes PYCR1/2 in vivo, both with organ culture and a knockout mouse line. While these in vivo studies did not show a gross phenotype associated with defective embryonic development, the authors conclude that a tightly controlled level of pyruvate and PYCR1/2 activity is necessary to prevent premature differentiation of nephron progenitors.

Comments for the author

The authors have addressed all my concerns and the manuscript is much improved.

Reviewer 2

Advance summary and potential significance to field

The manuscript by Kwon et al addressed the role of metabolism in renal development. As this area is so far very understudied, addressing this area is by itself very innovative and can advance the field.

Comments for the author

The authors have properly addressed my previous comments. The paper has clearly improved. However, in my opinion, the fact that metabolomics was carried out in a cell line and not in NP cells remains a problem.

© 2022. Published by The Company of Biologists under the terms of the Creative Commons Attribution License (https://creativecommons.org/licenses/by/4.0/).

The authors may find solutions to this problem in PMID: 26190145. Without performing metabolomics in bona fide NP cells the conclusions drawn need to be tuned down. In particular, the title referring to the MAPK/ERK pathway as a gatekeeper needs to be changed.

First revision

Author response to reviewers' comments

Reviewer 2 Advance summary and potential significance to field
The manuscript by Kwon et al addressed the role of metabolism in renal development. As this area is so far very understudied, addressing this area is by itself very innovative and can advance the field.

Comments for the author
The authors have properly addressed my previous comments. The paper has clearly improved. However, in my opinion, the fact that metabolomics was carried out in a cell line and not in NP cells remains a problem.
The authors may find solutions to this problem in PMID: 26190145. Without performing metabolomics in bona fide NP cells the conclusions drawn need to be tuned down. In particular, the title referring to the MAPK/ERK pathway as a gatekeeper needs to be changed.

Response: We thank the reviewer for providing very valuable comments that helped us to shape our manuscript into a what we think much-improved version. As pointed out by the reviewer, we have now modified the title of our work to more accurately describe our findings, which indeed derive from the experiments done both in cultured cells and in vivo nephron progenitors. In essence, we no longer refer MAPK/ERK pathway as a gatekeeper of nephron progenitor metabolism in the title, which now runs “Omics profiling discovers the regulatory functions of MAPK/ERK pathway in nephron progenitor metabolism”. We have accordingly tuned down our conclusions about the essential functions of MAPK/ERK pathway in nephron progenitor metabolism (new/changed text highlighted in blue). We hope that the reviewer finds these modifications adequate enough for endorsing publication of our results.

Second decision letter

MS ID#: DEVELOP/2022/200986

MS TITLE: Omics profiling discovers the regulatory functions of MAPK/ERK pathway in nephron progenitor metabolism

AUTHORS: Hyuk Nam Kwon, Kristen Kurtzeborn, Vladislav Iaroshenko, Xing Jin, Abigail Loh, Nathalie Escande-Beillard, Bruno REVERSADE, Sunghyouk Park, and Satu Kuure
ARTICLE TYPE: Research Article

Thank you for sending your manuscript to Development through Review Commons.

I am happy to tell you that your manuscript has been accepted for publication in Development, pending our standard ethics checks.

Reviewer 2

Advance summary and potential significance to field

Comments were adequately addressed.

Comments for the author

Comments were adequately addressed.

© 2022. Published by The Company of Biologists under the terms of the Creative Commons Attribution License (https://creativecommons.org/licenses/by/4.0/).
